# Arresting the Catalytic Arginine in Chlorite Dismutases: Impact on Heme Coordination, Thermal Stability, and Catalysis

**DOI:** 10.1021/acs.biochem.0c00910

**Published:** 2021-02-15

**Authors:** Daniel Schmidt, Ilenia Serra, Georg Mlynek, Vera Pfanzagl, Stefan Hofbauer, Paul G. Furtmüller, Kristina Djinović-Carugo, Sabine Van Doorslaer, Christian Obinger

**Affiliations:** † Department of Chemistry, Institute of Biochemistry, University of Natural Resources and Life Sciences, Vienna, Muthgasse 18, A-1190 Vienna, Austria; ‡ BIMEF Laboratory, Department of Chemistry, University of Antwerp, 2020 Antwerp, Belgium; § Department of Structural and Computational Biology, Max Perutz Laboratories, University of Vienna, A-1030 Vienna, Austria; ∥ Department of Biochemistry, Faculty of Chemistry and Chemical Technology, University of Ljubljana, Večna pot 5, SI-1000 Ljubljana, Slovenia

## Abstract

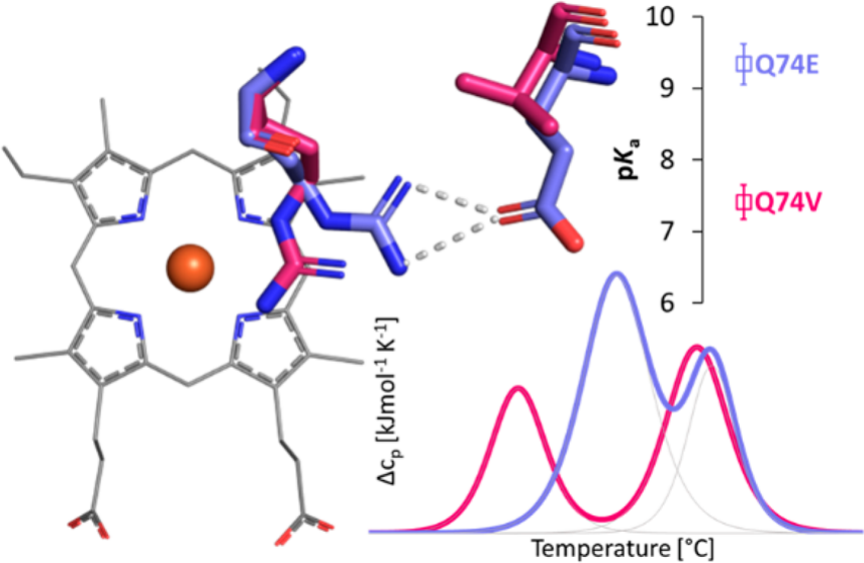

Chlorite dismutases (Clds) are heme *b*-containing oxidoreductases that can decompose chlorite to chloride and molecular oxygen. They are divided in two clades that differ in oligomerization, subunit architecture, and the hydrogen-bonding network of the distal catalytic arginine, which is proposed to switch between two conformations during turnover. To understand the impact of the conformational dynamics of this basic amino acid on heme coordination, structure, and catalysis, Cld from *Cyanothece* sp. PCC7425 was used as a model enzyme. As typical for a clade 2 Cld, its distal arginine 127 is hydrogen-bonded to glutamine 74. The latter has been exchanged with either glutamate (Q74E) to arrest R127 in a salt bridge or valine (Q74V) that mirrors the setting in clade 1 Clds. We present the X-ray crystal structures of Q74V and Q74E and demonstrate the pH-induced changes in the environment and coordination of the heme iron by ultraviolet–visible, circular dichroism, and electron paramagnetic resonance spectroscopies as well as differential scanning calorimetry. The conformational dynamics of R127 is shown to have a significant role in heme coordination during the alkaline transition and in the thermal stability of the heme cavity, whereas its impact on the catalytic efficiency of chlorite degradation is relatively small. The findings are discussed with respect to (i) the flexible loop connecting the N-terminal and C-terminal ferredoxin-like domains, which differs in clade 1 and clade 2 Clds and carries Q74 in clade 2 proteins, and (ii) the proposed role(s) of the arginine in catalysis.

Chlorite dismutases (Clds, EC 1.13.11.49) are heme *b*-containing oxidoreductases found exclusively in prokaryotic organisms.^1,2^ They belong to the structural superfamily of porphyrin-binding dimeric α + β barrel proteins that typically show a high degree of functional diversity.^3,4^ Clds convert chlorite (ClO_2_^–^) to chloride (Cl^–^) and dioxygen, therefore being of interest for biotechnological and bioremediation applications.^1,2^ The formation of an oxygen–oxygen bond during turnover constitutes an unusual biochemical reaction that otherwise is performed by only the water-splitting manganese complex of photosystem II in oxygenic organisms and an enzyme from an anaerobic methane-oxidizing bacterium.^5^

Chlorite dismutases are divided into two lineages denoted as clade 1 (“long Clds”) and clade 2 (“short Clds”). Figure 1 shows the X-ray crystal structures of two representative chlorite dismutases, namely, of the clade 1 Cld from *Candidatus* “*Nitrospira defluvii*” [*Nd*Cld, PDB entry 3NN1 (Figure 1)] and of the clade 2 Cld from *Cyanothece* sp. PCC7425 [*C*Cld, PDB entry 5MAU (Figure 1)], respectively. The two clades exhibit differences in (i) oligomerization, with clade 1 proteins being homopenta- or homohexamers^6−10^ and clade 2 representatives forming homodimers,^11−14^ and (ii) subunit structure, with “long” clade 1 Clds being composed of an N-terminal and a C-terminal heme *b*-carrying ferredoxin-like domain and “short” clade 2 proteins lacking the α-helices of the N-terminal domain (Figure 1). The C-terminal domain of clade 2 subunits is very similar to that of clade 1 enzymes, including the architecture of the heme cavity (Figure 1).^4,6,8,11,14^ This high degree of similarity is reflected by similar redox properties and chlorite degradation activities.^1,2,15^

**Figure 1:**
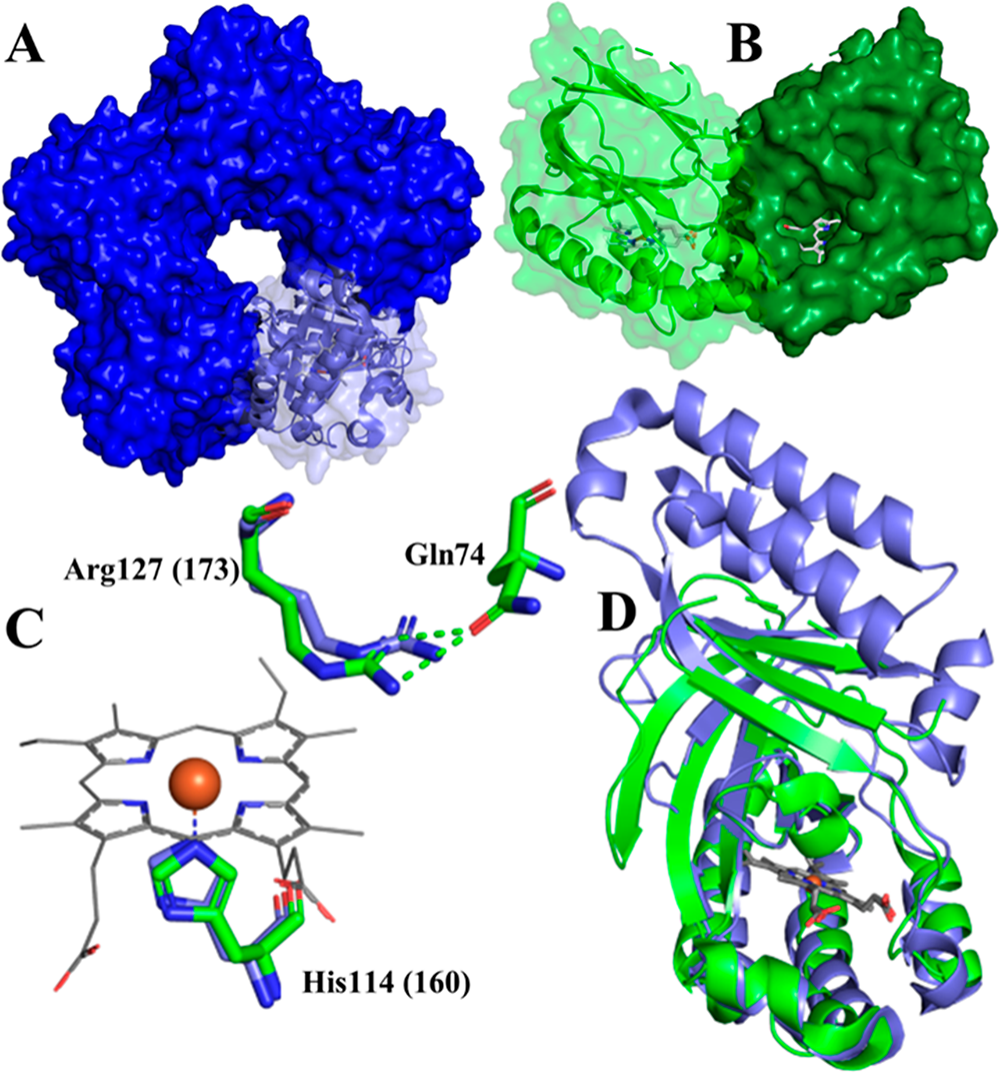
Structures of pentameric chlorite dismutase from *N. defluvii* (*Nd*Cld, PDB entry 3NN1)^8^ and dimeric chlorite dismutase from *Cyanothece* sp. PCC7425 (*C*Cld, PDB entry 5MAU).^14^ (A) Surface representation of the *Nd*Cld pentamer. One subunit is depicted in ribbon representation, with a transparent surface illustration. (B) Dimeric *C*Cld with one subunit shown in ribbon representation and a transparent surface illustration. Heme prosthetic groups are shown as gray sticks, and the metal ion is shown as an orange sphere. (C) Alignment of active site residues of *C*Cld (green) and *Nd*Cld (blue), focusing on the catalytically important arginine (R127 and R173). Amino acids are numbered according to *C*Cld and *Nd*Cld (brackets). The heme group (gray) is depicted in line representation. (D) Superimposition of subunits of *C*Cld (green) and *Nd*Cld (blue) in ribbon representation. This figure was generated using PyMOL (http://www.pymol.org/).

In the ferric resting state, the heme iron of Clds is ligated by a proximal histidine (H160 in *Nd*Cld and H114 in *C*Cld) and a distal water.^14^ The only charged amino acid in the hydrophobic distal heme cavity is a fully conserved arginine (R173 in *Nd*Cld and R127 in *C*Cld), which is proposed to be flexible and to switch between two conformations during catalysis, i.e., pointing toward either the heme iron (“in”) or the substrate entry channel (“out”).^4,6,8,11,14,16^ Importantly, the hydrogen-bonding network of this catalytic arginine is different in clade 1 and clade 2 Clds. In clade 2 Clds, R127 is H-bonded to Q74 located on a flexible α-helical loop (Figure 2 and Table S1), which in general connects the N-terminal and C-terminal ferredoxin-like domains in porphyrin-binding α + β barrel proteins.^4^ By contrast, in clade 1 Clds the corresponding residue [Q123 in *Nd*Cld (Table S1)] turns away from R173 due to a different conformation of this loop (Figure 2, loop colored blue). As a consequence, a valine comes close to R173 in *Nd*Cld.^9,11^

**Figure 2:**
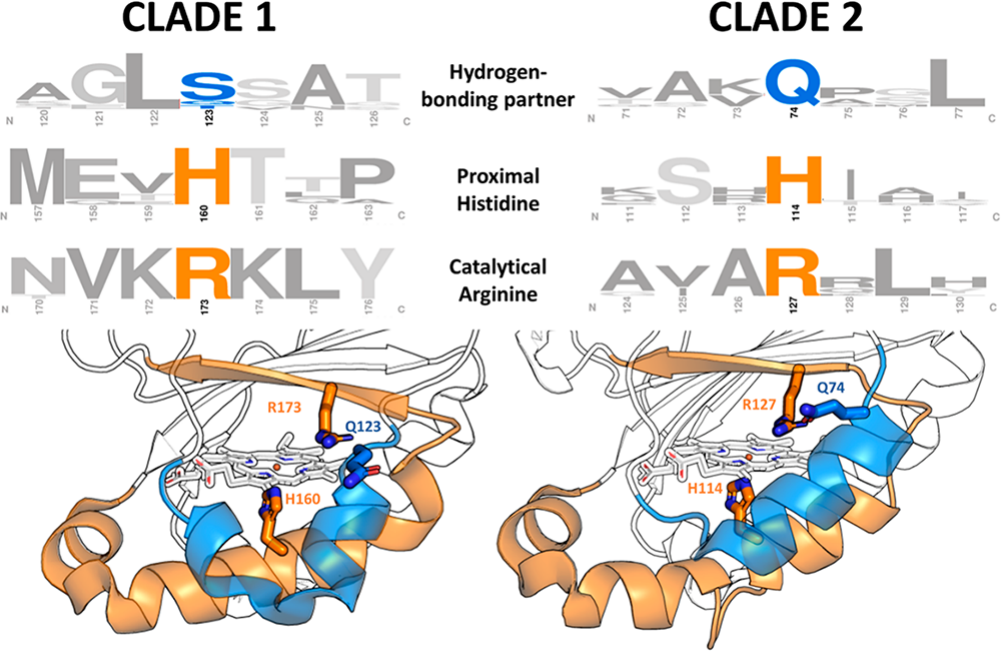
Comparison of the active site residues and architecture of clade 1 and clade 2 chlorite dismutases. Sequence alignments are represented as WebLogos. Structural differences are shown in cartoon representation, with highlighted amino acid residues depicted as sticks. The heme *b*-binding ferredoxin-like folds with catalytically important arginine (clade 1, R173; clade 2, R127) and proximal histidine (H160, H114) residues are colored orange. The linker region carrying the potential hydrogen-bonding partner of catalytic arginine (Q123 and Q74) is colored blue. This figure was generated using PyMOL (http://www.pymol.org/).

The conserved arginine has been the center of numerous structural and functional studies for its putative involvement in substrate recognition and/or in preventing irreversible enzyme inactivation.^9,16,17^ It has been postulated that this basic residue supports the (either heterolytic or homolytic) cleavage of chlorite and the recombination reaction between the postulated transient intermediates (hypochlorite and Compound I or chlorine monoxide and Compound II)^1,2,14,16^ and dictates the pronounced pH dependence of chlorite degradation.^17,18^

In the crystal structure of *C*Cld, in the resting state the heme iron participates in a distal H-bonding network that includes the metal ion, two water molecules (W501 and W502),^14^ R127 in the “out” conformation, and Q74.^14^ At alkaline pH, deprotonation of W501 shortens its distance to the heme iron and significantly weakens or even breaks the interaction between OH^–^ and W502 and simultaneously strengthens the interaction between W502 and R127.^14^ It was hypothesized that this alkaline transition might contribute to the pronounced pH dependence of chlorite degradation activity, which significantly decreases at alkaline pH.^1,2,7−9,13,14,17,18^

Here, we aimed to study the impact of the flexibility and the H-bonding network of R127 in *C*Cld by exchanging Q74 with valine (Q74V) or glutamic acid (Q74E). The Q74V variant mirrors the situation in the clade 1 representative *Nd*Cld and should exhibit an increased flexibility of R127 compared to that of wild-type *C*Cld, whereas Q74E might arrest the catalytic amino acid by forming a salt bridge. We provide the crystal structures of Q74V and Q74E and demonstrate the impact of the mutations and pH on heme coordination and active site architecture by ultraviolet–visible (UV–vis), electron paramagnetic resonance (EPR), and electronic circular dichroism (ECD) spectroscopy. Besides heme coordination, the mutations are shown to have a strong impact on the thermal stability of the heme cavity but an only weak effect on the chlorite degradation activity. The findings are discussed with respect to the proposed reaction mechanism(s) of the conversion of chlorite to chloride and dioxygen.

## Materials and Methods

### Sequence Alignment

Multiple-sequence alignments of clade 1 and 2 Clds were constructed using ClustalW^19^ (Table S1) with the following parameters: gap opening penalty of 10 and gap extension penalties of 0.20. All tools for sequence alignments were embedded in the MEGA X package.^20^ From these sequence alignments, sequence motifs were generated using WebLogo (http://weblogo.berkeley.edu/).

### Cloning, Site-Directed Mutagenesis, Expression, and Purification

To generate plasmids for protein expression, the existing plasmid (pET-52b^+^ vector) encoding the N-terminal HRV 3C-cleavable Strep(II)-tagged wild-type *C*Cld was used as a template.^13,14^ As a result of low-purification yields using the existing Strep(II) tag, the affinity tag was exchanged with a His tag using polymerase chain reaction (primers listed in Table S2) and NEBuilder HiFi DNA Assembly (New England Biolabs, Ipswich, MA). To confirm successful cloning, the final plasmid was sequenced from primer pET-up (Microsynth, Balgach, Switzerland). *C*Cld variants Q74E and Q74V were obtained by site-directed mutagenesis using the QuikChange Lightning Kit (Agilent, Santa Clara, CA). The modified wild-type plasmid described above was used as a template (Table S2).

Recombinant protein expression of wild-type *C*Cld and variants was performed in *Escherichia coli* BL21 Gold (DE3) cells (Agilent) in LB medium supplemented with ampicillin. The cells were incubated at 37 °C (180 rpm) until an OD_600_ of approximately 0.8 was reached. After the temperature had been decreased to 19 °C, protein expression was induced by adding 0.5 mM isopropyl β-d-thiogalactopyranoside and the cultures were kept at this temperature overnight. The cells were harvested by centrifugation (4 °C, 5000*g*, 20 min) and stored at −30 °C until further purification.

For protein purification, the cell pellet was thawed and resuspended in lysis buffer [50 mM phosphate buffer (pH 7.4), 500 mM NaCl, 0.5% Triton X-100, and 5% glycerol] with ∼100 μM hemin. Following two 3 min cycles of ultrasonication (pulsed mode, 1 s sonication, 1 s rest, 90%) on ice, the lysate was centrifuged (4 °C, 17000*g*, 35 min). The resulting supernatant was filtered (0.45 μm, Durapore Membrane, Merck, Darmstadt, Germany) before being loaded onto a His-trap affinity column (5 mL; GE Healthcare, Chicago, IL) pre-equilibrated with binding buffer [50 mM phosphate buffer (pH 7.4) and 500 mM NaCl]. After the loaded column had been washed with binding buffer, on-column cleavage of the His tag was performed by equilibrating with cleavage buffer (50 mM Tris-HCl with 150 mM NaCl and 1 mM EDTA) and cleaving with a His-tagged HRV 3C PreScission Protease overnight at 4 °C. Elution was carried out with storage buffer [50 mM phosphate buffer (pH 7.0)] accompanied by a concentration and desalting step using an Amicon Ultra-15 centrifugal filter unit (10 kDa molecular weight cutoff; Merck). As a final polishing step, the concentrated protein was applied to a pre-equilibrated [50 mM phosphate buffer (pH 7.0)] HiLoad 16/60 Superdex 200 prep grade column (GE Healthcare). The collected fractions were pooled, concentrated to ∼20 mg mL^–1^ using a centrifugal filter unit, and stored at −80 °C in 50 μL aliquots. Protein expression and purification of *Nd*Cld were performed as reported previously.^9^

### UV–vis and ECD Spectroscopy

UV–vis spectra in a wavelength range between 200 and 700 nm were recorded routinely using a Cary 60 UV–vis spectrophotometer (Agilent) and a model U-3900 spectrophotometer (Hitachi, Mannheim, Germany) at 25 °C. The molar extinction coefficient of heme (ε_Soret_ = 100000 M^–1^ cm^–1^) was used to determine the enzyme concentration. To determine the p*K*_a_ for the alkaline transition, 200 μL of a 10 μM enzyme solution in 5 mM sodium phosphate buffer (pH 7.0) was mixed with the same volume of 100 mM buffer at the desired pH value (citrate-phosphate buffer, pH 4–6; phosphate buffer, pH 6–9; borate-phosphate buffer, pH 9–12). Absorption values at specific wavelengths reflecting peaks in difference spectra at acidic and alkaline pH regimes (i.e., 404, 406, 415, 423, 456, 508, 575, and 641 nm) were plotted against pH values. Sigmaplot (version 13.0, Systat Software, San Jose, CA) was used to fit the resulting curve to the following sigmoidal equation with *x*_0_ corresponding to p*K*_a_, *x* being the pH of the buffer, and *y* being the absorption at the observed wavelength:
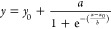


Electronic circular dichroism spectroscopy was performed using Chirascan (Applied Photophysics, Leatherhead, U.K.). Spectra were recorded in the far-UV region (180–260 nm) and in the visible region (260–500 nm) using 10 μM *C*Cld. The path lengths for the far-UV and visible region were 1 and 10 mm, respectively. Used buffers were 5 mM citrate-phosphate buffer (pH 5.0), 5 mM phosphate buffer (pH 7.0), and 5 mM borate-phosphate buffer (pH 9.0). The spectral bandwidth was set to 1 mm, and the scan speed was 5 s nm^–1^.

Temperature-mediated unfolding between 30 and 70 °C was monitored using a heat rate of 1 °C min^–1^. Single-wavelength scans were performed in the far-UV (210 nm) and visible regions (Soret maximum). For data processing, Pro-Data Viewer provided by Applied Photophysics was used. The fraction of unfolded protein (α) was calculated according to the formula α = (θ_N_ – θ)/(θ_N_ – θ_U_), with θ being the ellipticity of the protein at a distinct temperature (*T*), θ_N_ being the ellipticity (in millidegrees) at 210 nm or the Soret maximum (between 411 and 422 nm, depending on the pH value) of the native protein, and θ_U_ representing the ellipticity at 210 nm or the Soret maximum of the unfolded protein.

### Electron Paramagnetic Resonance Spectroscopy

Continuous-wave (CW) EPR spectroscopy measurements were performed to assess the spin state of ferric wild-type forms of *Nd*Cld and *C*Cld as well as the Q74V and Q74E variants and to investigate pH-induced changes in the local environment of the heme iron. To maintain the consistency with other spectroscopic and biochemical assays, the EPR samples of *Nd*Cld and *C*Cld were prepared in the same (or most similar) buffers used for the other experiments described in this work. Samples of wild-type *Nd*Cld were prepared at a concentration of 400 μM in 50 mM HEPES buffer (pH 7.0), in 50 mM MES buffer (pH 5.5), and in 50 mM borate buffer (pH 10.0). Solutions of wild-type *C*Cld, Q74V, and Q74E were prepared in a 50 mM sodium phosphate-citrate buffer mixture (pH 5.0), 50 mM sodium phosphate (sodium phosphate monobasic-dibasic mixture) (pH 7.0), 50 mM sodium-phosphate-borate buffer mixture (pH 9.0), and 50 mM borate buffer (pH 10.0) at a concentration of ∼400 μM. To check the effect of a potential pH change upon freezing on EPR signatures, the EPR spectra of wild-type *Nd*Cld and *C*Cld at pH 7 were also recorded in a “temperature-independent” buffer (45% HEPES and 55% sodium phosphate^21^), and no significant changes were observed (data not shown). The influence of the cryoprotectant glycerol on the EPR spectra of wild-type *Nd*Cld and *C*Cld was tested at neutral pH. In the quartz EPR tubes (diameter of 4 mm) containing the samples, the excess of paramagnetic O_2_ was removed in several freeze–pump–thaw cycles prior to the starting of the EPR experiment and all tubes were vacuum-pumped during the measurements.

X-Band CW-EPR experiments were performed on a Bruker ESP300E spectrometer equipped with a liquid helium cryostat (Oxford Inc.) and operating at a microwave frequency of ∼9.44 GHz. Spectra were recorded at 10 K, under nonsaturating conditions at microwave powers in the range of 2–5 mW, a modulation frequency of 100 kHz, and a modulation amplitude of 1 mT. Simulations of experimental spectra were performed with EasySpin software implemented in Matlab.^22^

### Differential Scanning Calorimetry

Differential scanning calorimetry (DSC) experiments were performed on a MicroCal PEAQ-DSC Automated instrument (Malvern Panalytical Ltd., Malvern, U.K.) equipped with an autosampler for 96-well plates and controlled by the MicroCal PEAQ-DSC software (cell volume of 130 μL). Samples were analyzed over a temperature range of 20–80 °C, using a heating scan rate of 60 °C h^–1^. Each sample was immediately rescanned to check for reversible unfolding. Furthermore, if no refolding was detectable, this rescan was used as a baseline. *C*Cld variants were applied as a 20 μM solution in the range from pH 5.0 to 9.0. For the pH dependence measurements of thermal unfolding, 50 mM citrate phosphate buffer (pH 5.0–6.0) and 50 mM phosphate buffer (pH 6.0–9.0) were used. Fitting of the data was performed with the MicroCal PEAQ-DSC software using a non-two-state equilibrium unfolding model.

### X-ray Crystallography

Crystallization experiments were performed using the sitting drop vapor diffusion method in SWISSCI MRC three-well crystallization plates (Molecular Dimensions, Newmarket, U.K.). Crystallization drops were set up using a mosquito crystallization robot (TTP Labtech). The reservoir was filled with 40 μL of a precipitant solution. In the sample wells, protein:precipitant ratios of 150:200, 200:200, and 250:200 were dispensed. The protein concentration was approximately 10 mg/mL in 50 mM sodium phosphate buffer (pH 7.0). In addition to known crystallization conditions [0.1 M MES (pH 6.5), 0.15 M MgSO_4_, 28% (w/v) polyethylene glycol 3350, and 3% (v/v) glycerol].^14^ Commercially available crystallization screens were used for further screening. Crystallization plates were stored in a Formulatrix RI-1000 imaging device at 22 °C. Successful hits were obtained using the known conditions (Q74E) and the SG1 Screen from Molecular Dimensions (Q74V). Initial screening conditions were optimized for the growth of larger crystals by using the microseeding technique. For this method, a single crystal was placed in a crystallization solution in a fresh tube and crushed by vortexing using the Seed Bead kit (Hampton Research, Aliso Viejo, CA). The seeding solution was used in a ratio of 1:100 for further crystallization. Final conditions were as follows: 0.1 M Tris (pH 8.5), 0.2 M MgCl_2_, and 20% (w/v) PEG 8000 for Q74V and 0.1 M MES (pH 6.5), 0.15 M MgSO_4_, 28% (w/v) PEG 3350, and 3% (v/v) glycerol for Q74E. Crystals were soaked with mother liquor supplemented with 20% (w/v) glycerol, harvested using a cryo-loop, and flash-vitrified in liquid nitrogen. Data were collected at 100 K using a PILATUS 6M detector (25 Hz, 450 μm sensor thickness) at beamline P13 operated by EMBL Hamburg at the PETRA III storage ring (DESY, Hamburg, Germany).^23^

Data sets were processed with XDS, and symmetry equivalent reflections merged with XDSCONV.^24^ Intensities were not converted to amplitudes. Initially, a conservative high-resolution cutoff *I*/σ of 1–2 was used.^25^ The phase problem was determined by molecular replacement using phenix.phaser^26^ taking the search model of PDB entry 5MAU.^14^ The model was further improved by iterative cycles of manual model building using COOT^27^ and maximum likelihood refinement using phenix.refine.^28^ Phenix.refine converted intensities into amplitudes using the French and Wilson algorithm.^29^ The *B*-factor model was selected using the algorithm implemented in PDB_REDO, which is based on the number of X-ray reflections per atom and a recent implementation of the Hamilton test.^30−32^ The final high-resolution cutoff was based on performing paired refinement using the PDB_REDO Web server.^33^ Final stages of refinement included either translation liberation screw (TLS) parameters, isotropic, or anisotropic *B*-factor models, automated addition of hydrogens and water molecules, optimization of X-ray/ADP weight, and optimization of X-ray/stereochemistry weight. The model was validated with MolProbity.^34^ The figures were prepared with PyMOL Molecular Graphics System (version 2.3.4, Schrödinger, LLC). Atomic coordinates have been deposited in the Protein Data Bank as entries 7ATI (*C*Cld Q74V) and 7ASB (*C*Cld Q74E).

### Chlorite Degradation Activity

Enzyme-mediated chlorite degradation was measured polarographically following the release of O_2_ by using a Clark-type oxygen electrode (Oxygraph Plus; Hansatech Instruments, Norfolk, U.K.). Reactions were monitored at 30 °C using a connected water bath. The electrode was calibrated by equilibrating to 100% O_2_ saturation by bubbling with air and to 0% O_2_ saturation by flushing with N_2_ until stable plateaus were reached to derive an offset and calibration factor. Reactions were performed in O_2_-free 50 mM sodium phosphate buffer with pH values ranging from 5.0 to 9.0. The substrate was added to final concentrations from 20 μM to 1 mM NaClO_2_. Concentrations of the chlorite stock solutions were measured using a molar extinction coefficient at 260 nm of 154 M^–1^ cm^–1^.^35,36^ Subsequently, the reaction was started by adding 20 nM *C*Cld. Molecular oxygen production rates (micromolar O_2_ per second) were determined from the initial linear time traces and plotted against chlorite concentration. Furthermore, the total produced O_2_ (micromolar) was obtained by measuring the oxygen concentration until formation of a stable plateau after several minutes, depending on the *C*Cld variant and the chlorite concentration.

### Data Availability

The structures presented in this paper have all been deposited in the PDB as entries 7ASB (Q74E) and 7ATI (Q74V). All remaining data are included herein.

## Results

### Electronic Configuration of Ferric Wild-Type *C*Cld and Variants Q74V and Q74E

The far-UV ECD spectra (200–260 nm) of wild-type *C*Cld and the two variants are very similar (ellipticity minimum at ∼210 nm), suggesting that the overall secondary structure has not been altered by the inserted mutations (Figure S1A). Increasing the pH from 5 to 9 had no impact on the spectral characteristics.

**Figure 3:**
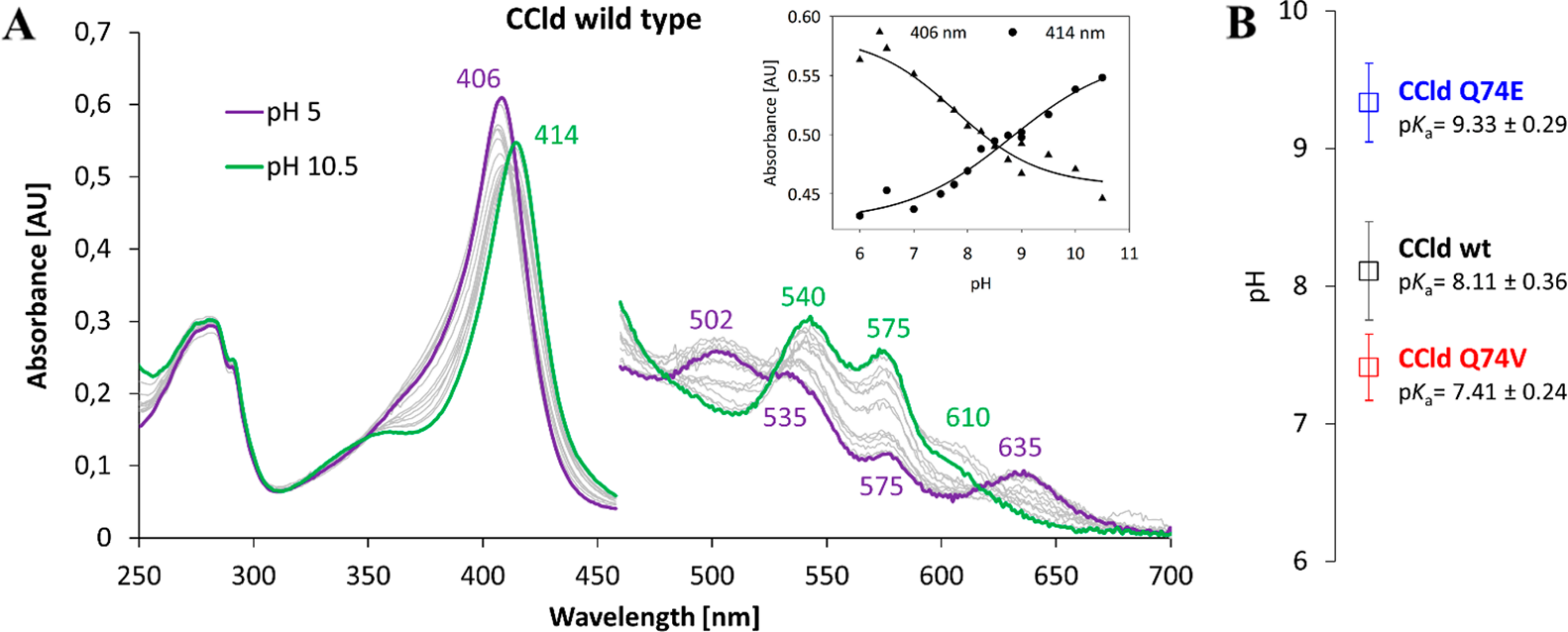
pH-dependent UV–vis spectral changes of wild-type *C*Cld and variants Q74V and Q74E. (A) UV–vis spectra of wild-type *C*Cld in the pH range of 5.0–10.5. The enzyme concentration was 5 μM. Very similar spectra were obtained for both variants (Figure S2). For better visualization of the CT bands and Q-bands, the spectra in the wavelength range between 460 and 700 nm are magnified 6-fold. The inset shows an increase and a decrease in absorbance at 406 and 414 nm, respectively, with a change in pH. The sigmoidal fit is shown as a black line. (B) Differences in p*K*_a_ values of the alkaline transition between wild-type *C*Cld and variants.

Both Q74V and Q74E show UV–vis spectral characteristics at acidic pH (i.e., pH 6.0) similar to those of the wild-type protein (Figure 3A, purple solid line; Figure S2). The ferric high-spin state is characterized by a Soret band at 406 nm, Q-bands at 502, 535, and 575 nm, and a charge transfer band (CT) at 635 nm. Under more alkaline conditions, a distinct shift to a low-spin form is observable. The Soret band is shifted to 414 nm, and Q-bands at 540 and 575 nm and a weak CT band at 610 nm appear (Figure 3A, green solid line; Figure S2). These spectral properties are found in all *C*Cld variants investigated in this study and are in agreement with previously reported data for the wild-type enzyme.^13,14^ Interestingly, while the spectral changes of the observed alkaline transition (i.e., deprotonation of distal water) are similar, the calculated p*K*_a_ values differ significantly from that of wild-type *C*Cld (p*K*_a_ = 8.11 ± 0.36) with a p*K*_a_ of 7.41 ± 0.24 for Q74V and a p*K*_a_ of 9.33 ± 0.29 for Q74E (Figure 3B).

Similar to the UV–vis experiments, the ECD spectra in the visible region strongly depend on pH (Figure S1B). The Soret bands exhibit a positive ellipticity with similar maxima at pH 5.0 (wild type, 414 nm; Q74V, 413 nm; Q74E, 412 nm). With both wild-type *C*Cld and Q74V, the Soret maxima are red-shifted with an increase in pH (pH 7, 415 and 416 nm, respectively; pH 9, 422 and 423 nm, respectively). By contrast, the shift is less pronounced in the Q74E variant (pH 7.0, 412 nm; pH 9.0, 415 nm).

Next, the impact of the altered hydrogen-bonding network of distal arginine with particular attention to pH-induced changes was probed by comprehensive EPR analysis and novel information about the amino acid environment at the heme site in clade 2 Clds is provided. The low-temperature CW-EPR spectra of *Nd*Cld and *C*Cld at neutral pH have been reported previously.^13,15,16^

In high-spin (*S* = ^5^/_2_) ferric heme proteins, a very large zero-field splitting (ZFS) is responsible for the typical CW-EPR spectral shape observed at X-band. Because the ZFS is much larger than the photon energy of the microwaves used, only transitions in the first Kramers doublet (*m*_s_ = ±^1^/_2_) can be detected. For this reason, EPR spectra of these proteins can be simulated either as effective *S* = ^1^/_2_ systems, using a **g**^**eff**^ tensor, where *g*_*x*_^eff^ and *g*_*y*_^eff^ are symmetrically split at ∼6 and *g*_*z*_^eff^ is close to 2, or as real *S* = ^5^/_2_ systems, where the **g** tensor is almost isotropic with principal values close to 2, and the observed features depend essentially on the *E*/*D* ratio between the rhombic (*E*) and tetragonal (*D*) zero-field splitting parameters.^37^ In this study, both simulation methods were used to obtain a complete picture of the systems under investigation. If a strong base is present at the sixth distal coordination position, the iron spin converts to the low-spin state (*S* = ^1^/_2_). In this case, the principal *g* values can be taken as a fingerprint and can be compared with those of other hexacoordinated heme systems in an attempt to identify the nature of the distal ligand.

**Figure 4:**
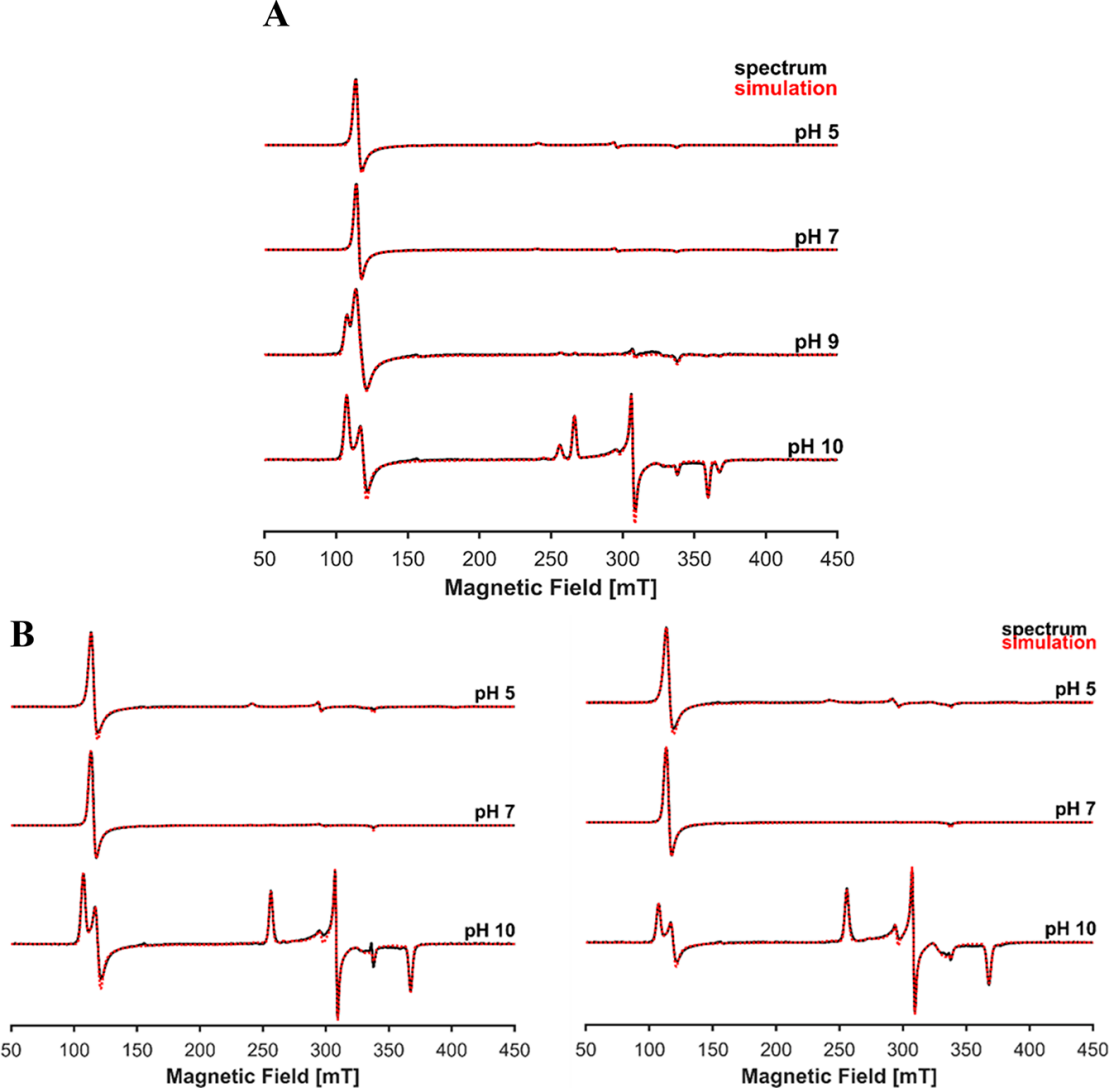
pH dependence of the CW-EPR spectrum of wild-type *C*Cld and variants Q74V and Q74E. Samples were prepared in 50 mM phosphate-citrate buffer (pH 5.0), 50 mM phosphate buffer (pH 7.0), 50 mM phosphate-borate buffer (pH 9.0), and 50 mM borate buffer (pH 10.0). A microwave power of 2 mW was applied to record the spectra. Solid black for the experimental spectrum, dashed red for the simulation. (A) Wild-type *C*Cld and (B) Q74E (left) and Q74V (right).

The EPR spectra of wild-type *C*Cld at different pH values are shown in Figure 4A. In a manner consistent with the published results mentioned above, the spectrum of *C*Cld at pH 7.0 is composed of two components due to high-spin heme centers (HS1 and HS2) and a smaller low-spin contribution of uncertain origin (LS1) (EPR parameters used for the simulation are reported in Table 1). The most dominant spectral contribution (HS1) is characterized by a near-axial effective **g** tensor (*g*_*x*_^eff^ ≈ *g*_*y*_^eff^) and concomitant very small *E*/*D* value. Notably, in the case of *Nd*Cld, the contributions due to high-spin hemes account for only ∼23% of the spectrum, which is instead dominated by a highly anisotropic low-spin signal (Table S3; simulations shown in Figure S3). No significant variations are observed when *C*Cld is brought to acidic pH (Figure 4A, top), while a trend becomes evident when the pH is increased to 10 (Figure 4A, bottom). An alkaline transition is partially observed at pH 9.0, when the appearance of new low-spin features is accompanied by a change in the high-spin component, from axial to rhombic **g**^eff^ tensor. At pH 10.0, two main low-spin species, denoted as LS1-alkaline and LS2-alkaline, are nicely resolved and the leftover high-spin (∼25%) is found to be completely rhombic (HS3). The principal *g* values of the low-spin components are typical of an OH^–^ coordination of the heme iron at the sixth position on the distal site.^12,38−40^

**Table 1:** EPR Simulation Parameters of Wild-Type *C*Cld at Different pH Values^a^

	species	*g_x_*^eff^	*g*_*y*_^eff^	*g*_*z*_^eff^	*g_x_*	*g*_*y*_	*g*_*z*_	*E*/*D*	%
pH 5	HS1	5.92	5.84	2.00	1.96	1.96	2.00	0.002	53
	HS2	6.09	5.68	1.99	1.96	1.96	2.00	0.009	19
	LS1				1.67	2.28	2.80	–	28
pH 7	HS1	5.90	5.86	2.00	1.96	1.96	2.00	<0.001	63
	HS2	6.09	5.68	2.00	1.96	1.96	2.00	0.009	17
	LS1				1.66	2.28	2.81	–	20
pH 9	HS1	5.90	5.86	2.00	1.96	1.96	2.00	<0.001	13
	HS2	6.03	5.69	2.00	1.95	1.95	2.00	0.007	22
	HS3	6.28	5.65	1.99	1.99	1.99	2.00	0.013	47
	LS1-alkaline				1.88	2.26	2.53	–	7
	LS2-alkaline				1.83	2.19	2.63	–	11
pH 10	HS3	6.29	5.66	1.99	1.99	1.99	2.00	0.013	25
	LS1-alkaline				1.87	2.20	2.53	–	46
	LS2-alkaline				1.83	2.19	2.63	–	23
	LS3-alkaline				1.80	2.27	2.75	–	6

a Errors in *g* values of ±0.01, errors in *E*/*D* ratios of ±0.001, and errors in contributions of ±1%.

At pH 7.0, the EPR spectra of variants Q74E and Q74V largely resemble that of the wild-type protein (Figure 4 and Figure S4) and can be simulated with similar parameters (Table 2). In addition, Q74E shows the presence of small contributions from three different low-spin components, while in the spectrum of Q74V, a single weak low-spin signal is barely observed (Figure S4). Remarkably, when the two proteins are measured at pH 5.0 (Figure 4B, top), a contribution of the same low-spin species found in the wild-type protein is detected. In all three proteins, the spectral contribution of this species accounts for ∼30% of the total EPR spectrum. At pH 10.0, the dominant low-spin component in the variants has the same *g* values as LS2-alkaline, while this was less abundant than LS1-alkaline in the wild-type case (Figure 4B and Figure S5). Furthermore, similar to wild-type *C*Cld, an axial-to-rhombic conversion occurs for the high-spin state of both Q74E and Q74V when the pH is increased (Figure 4B). Overall, these data indicate that the site-directed mutation of the glutamine does not substantially perturb the spin state of the heme iron in the resting state of *C*Cld. In addition, the EPR findings corroborate the pH-dependent trend observed by UV–vis spectroscopy and resonance Raman spectroscopy of wild-type *C*Cld.^14^

**Table 2:** EPR Simulation Parameters of Variants Q74E and Q74V of *C*Cld at Different pH Values^a^

	species	*g*_*x*_^eff^	*g*_*y*_^eff^	*g*_*z*_^eff^	*g*_*x*_	*g*_*y*_	*g*_*z*_	*E*/*D*	%
Q74E									
pH 5	HS1	5.92	5.83	2.00	1.96	1.96	2.00	0.002	48
	HS2	6.14	5.69	2.00	1.97	1.97	2.00	0.010	22
	LS1				1.67	2.28	2.79	–	30
pH 7	HS1	5.91	5.89	2.00	1.96	1.96	2.00	0.002	68
	HS2	6.16	5.68	2.00	1.97	1.97	2.00	0.010	13
	LS1				1.66	2.28	2.81	–	7
	LS2				1.81	2.27	2.62	–	8
	LS3				1.89	2.27	2.45	–	4
pH 10	HS3	6.29	5.66	1.99	1.99	1.99	2.00	0.013	24
	LS1-alkaline				1.88	2.20	2.53	–	1
	LS2-alkaline				1.83	2.19	2.63	–	65
	LS3-alkaline				1.82	2.27	2.75	–	10
Q74V									
pH 5	HS1	5.93	5.84	2.00	1.96	1.96	2.00	0.002	40
	HS2	6.15	5.68	1.99	1.97	1.97	2.00	0.010	23
	LS1				1.68	2.30	2.79	–	29
	LS2				1.89	2.28	2.72	–	8
pH 7	HS1	5.91	5.89	2.00	1.97	1.97	2.00	<0.001	81
	HS2	6.16	5.68	2.00	1.97	1.97	2.00	0.010	16
	LS3				1.89	2.27	2.45	–	3
pH 10	HS3	6.29	5.66	1.99	1.99	1.99	2.00	0.013	14
	LS2-alkaline				1.83	2.19	2.64	–	75
	LS3-alkaline				1.80	2.28	2.75	–	11

a Errors in *g* values of ±0.01, errors in *E*/*D* ratios of ±0.001, errors in contributions of ±1%.

It is interesting to note that in the alkaline spectrum of *Nd*Cld (Figure S3) the major low-spin species (excluding that already present at neutral pH) is characterized by *g* values close to those of LS2-alkaline (Table S3). This similarity with the Q74 *C*Cld variants is coherent with the absence of the conserved glutamine in *Nd*Cld, which would suggest that the positioning of the arginine has a role in determining the binding mode of hydroxide.

**Figure 5:**
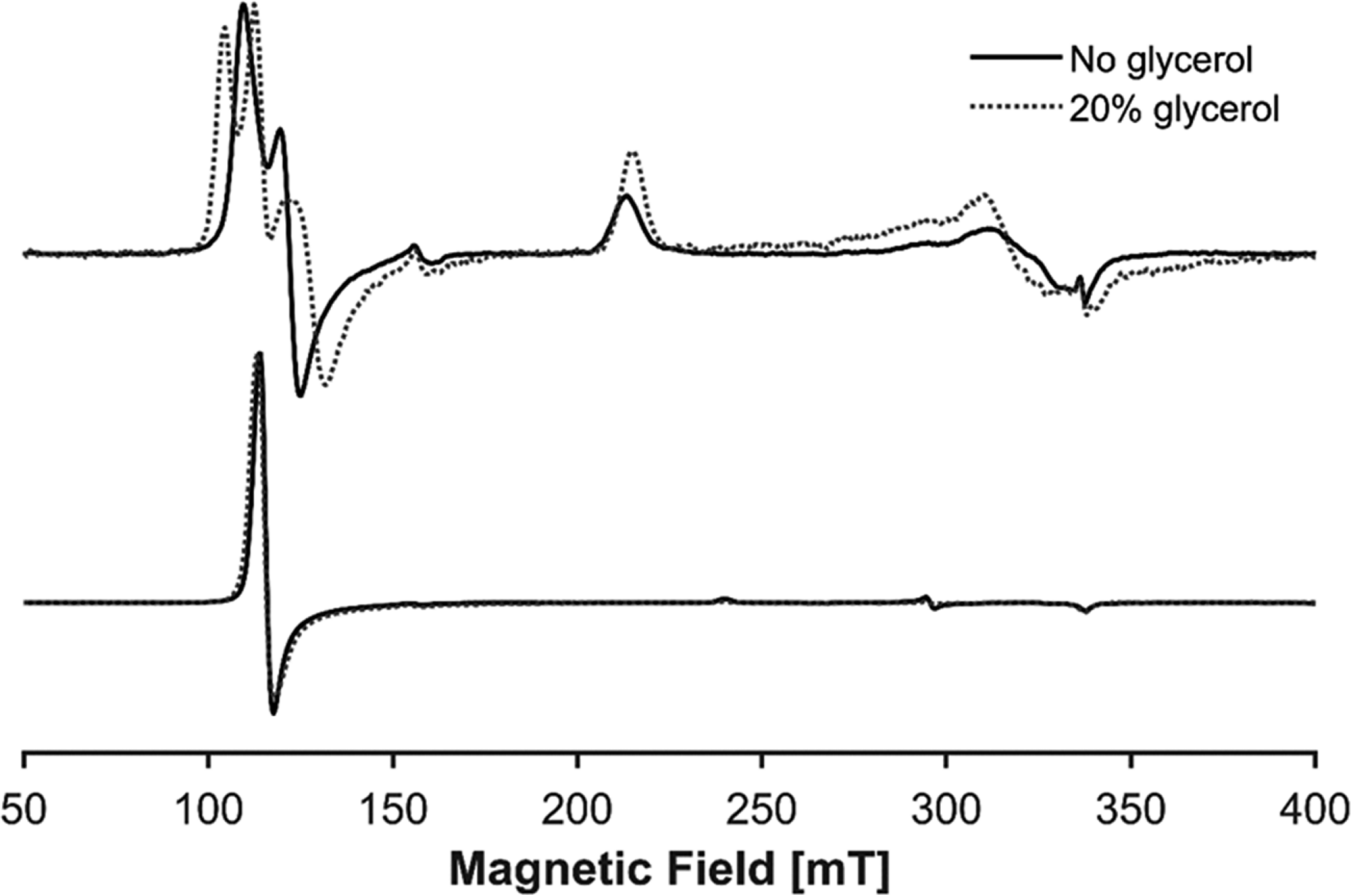
Effects of glycerol on the EPR spectra of wild-type *Nd*Cld and *C*Cld at neutral pH. Samples were prepared in 50 mM HEPES buffer (pH 7.0) (*Nd*Cld, top) and 50 mM phosphate buffer (pH 7.0) (*C*Cld, bottom). A microwave power of 5 mW was applied to record the spectra of *Nd*Cld, while a microwave power of 2 mW was used for *C*Cld spectra. Solid black for the sample without glycerol, dashed gray for the sample with 20% glycerol.

Finally, the restricted flexibility of the arginine in wild-type *C*Cld, compared to that of *Nd*Cld, might also be responsible for the different behavior of the two proteins in the presence of glycerol, at least at neutral pH. As depicted in Figure 5, the addition of 20% glycerol causes dramatic changes in the low-field part of the spectrum of *Nd*Cld (contributions of high-spin species), which now reveals a species with increased rhombicity and a well-resolved axial component. On the contrary, the spectrum of *C*Cld is scarcely affected by this compound, suggesting that the active site of this enzyme is less prone to accommodating the glycerol molecule. Preliminary results on the effects of glycerol at higher pH values (data not shown) indicate a strong influence of pH in the reactivity of chlorite dismutases with this compound; however, this aspect will be explored more in detail in the future.

### Thermal Stability of Wild-Type *C*Cld and Variants Q74V and Q74E

**Figure 6:**
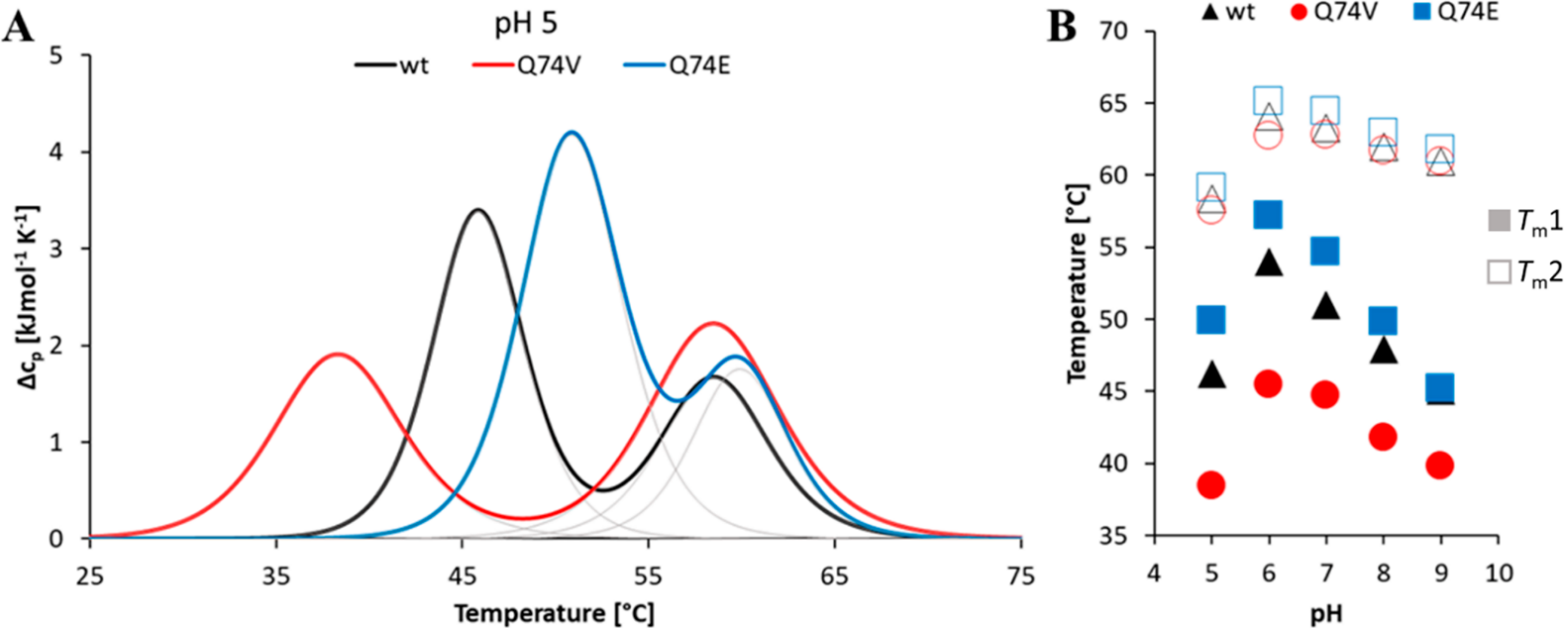
Thermal stability of wild-type *C*Cld and variants measured at different pH values by differential scanning calorimetry. The enzyme concentration was 20 μM. (A) Normalized thermograms of wild-type *C*Cld (black), Q74V (red), and Q74E (blue) at pH 5.0. Colored lines represent experimental data, and gray lines show a fit of the endotherm to a non-two-state transition model. (B) Calculated *T*_m_1 (filled symbols) and *T*_m_2 (empty symbols) plotted vs pH values. Measurements were performed in 50 mM citrate phosphate buffer (pH 5.0–6.0) and 50 mM phosphate buffer (pH 7.0–9.0).

Next, to investigate the influence of the introduced mutations and pH on protein stability, DSC and temperature-dependent ECD spectroscopy were performed. Figure 6 compares the thermograms of wild-type *C*Cld and variants at pH 5.0. Two distinct endotherms were observed, suggesting a non-two-state transition (*T*_m_1 and *T*_m_2) as described previously.^41^ In the pH regime of 5.0–9.0, the second transition (*T*_m_2) is at ∼58 °C for all three proteins, while significant differences were observed in *T*_m_1 values, ranging from 38.3 °C (Q74V) to 50.9 °C (Q74E) (Figure 6). Both wild-type *C*Cld and variants show the highest thermal stability at pH 6.0 (i.e., *T*_m_1 = 45.5–57.2 °C; *T*_m_2 = 62.7–65.1 °C) and a continuously decreasing thermal stability at more alkaline pH (Figure 6).

The pH dependence of thermal stability was also investigated by ECD in the far-UV and visible region at pH 5.0, 7.0, and 9.0 to assign the observed transitions to distinct unfolding events. Similar to DSC, two transitions were observed with similar *T*_m_ values (Table S4). ECD in the visible region clearly correlates with the first unfolding event and heme release due to disappearance of Soret ellipticity (411–423 nm), whereas the second endotherm reflects melting of the secondary structures due to the loss of ellipticity at 210 nm.

### X-ray Crystal Structures of Variants Q74V and Q74E

The X-ray crystal structure of ferric wild-type *C*Cld was already determined under various conditions, i.e., at pH 6.5 and 8.5, and with soaked-in distal ligands (F^–^ and SCN^–^) at pH 6.5.^14^ In all available *C*Cld structures, the (putatively) flexible arginine (R127) is present in the “out” conformation, i.e., pointing toward the substrate entry channel. This conformation is stabilized by a hydrogen bond to glutamine 74, which is in the proximity of R127 (2.85 Å).^14^

Variants Q74V and Q74E crystallized in space groups *P*12_1_1 and *P*1, respectively, with one dimer per asymmetric unit. Each subunit of the homodimeric proteins binds one heme *b*. Similar to that of wild-type *C*Cld,^14^ the structure consists of a ferredoxin-like fold that is defined by five α-helices along with a β-barrel composed of three-stranded and five-stranded antiparallel β-sheets. The crystals diffracted to high resolutions of 1.51 Å (Q74V) at pH 8.5 and 1.40 Å (Q74E) at pH 6.5 (Table 3). Figure S6 shows a comparison of the 2*F*_o_ – *F*_c_ electron density maps of the active sites of variants Q74V and Q74E contoured at σ = 1.5. The high degree of similarity with the overall structure of wild-type *C*Cld^14^ is reflected by root-mean-square deviation (rmsd) values of 0.253 Å (over 294 C_α_ atoms) and 0.055 Å (over 330 C_α_ atoms) for Q74V and Q74E, respectively.

**Table 3:** Crystallization Conditions and Data Collection and Refinement Statistics for *C*Cld Variants Q74V (PDB entry 7ATI) and Q74E (PDB entry 7ASB)^a^

	*C*Cld Q74V	*C*Cld Q74E
wavelength (Å)	0.9763	0.9763
resolution range (Å)	29.64–1.51 (1.564–1.51)	30.98–1.4 (1.45–1.4)
space group	*P*12_1_1	*P*1
unit cell dimensions	55.014 Å, 72.676 Å, 60.169 Å, 90°, 112.733°, 90°	51.16 Å, 52.738 Å, 54.772 Å, 107.41°, 99.143°, 108.984°
total no. of reflections	457965 (42914)	325936 (32812)
no. of unique reflections	68449 (6796)	90357 (8896)
multiplicity	6.7 (6.3)	3.6 (3.7)
completeness (%)	98.28 (84.44)	92.53 (91.39)
mean *I*/σ(*I*)	11.45 (0.35)	10.09 (1.14)
Wilson *B*-factor (Å^2^)	25.32	17.09
*R*_merge_	0.09328 (4.026)	0.06724 (1.195)
**R*_meas_*	0.1012 (4.39)	0.07945 (1.396)
**R*_pim_*	0.03886 (1.721)	0.04186 (0.7172)
*CC_1/2_*	0.999 (0.169)	0.998 (0.571)
CC*	1 (0.537)	0.999 (0.852)
no. of reflections used in refinement	67369 (5762)	90301 (8887)
no. of reflections used for *R*_free_	1575 (132)	1384 (151)
*R*_work_	0.1738 (0.4070)	0.1637 (0.3323)
*R*_free_	0.1956 (0.3953)	0.1825 (0.3423)
CC (work)	0.972 (0.489)	0.966 (0.804)
CC (free)	0.965 (0.268)	0.940 (0.710)
no. of non-hydrogen atoms	3470	3673
macromolecules	3065	3057
ligands	136	173
solvent	269	443
protein residues	364	362
rmsd for bonds (Å)	0.01	0.023
rmsd for angles (deg)	0.94	2.18
Ramachandran favored (%)	97.22	97.49
Ramachandran allowed (%)	2.78	2.23
Ramachandran outliers (%)	0	0.28
Rotamer outliers (%)	0	0
Clashscore	1.41	6.69
average *B*-factor (Å^2^)	35.7	25.31
macromolecules	35.47	23.44
ligands	37.56	30.4
solvent	37.38	36.26
no. of TLS groups	19	

a Statistics for the highest-resolution shell are shown in parentheses.

**Figure 7:**
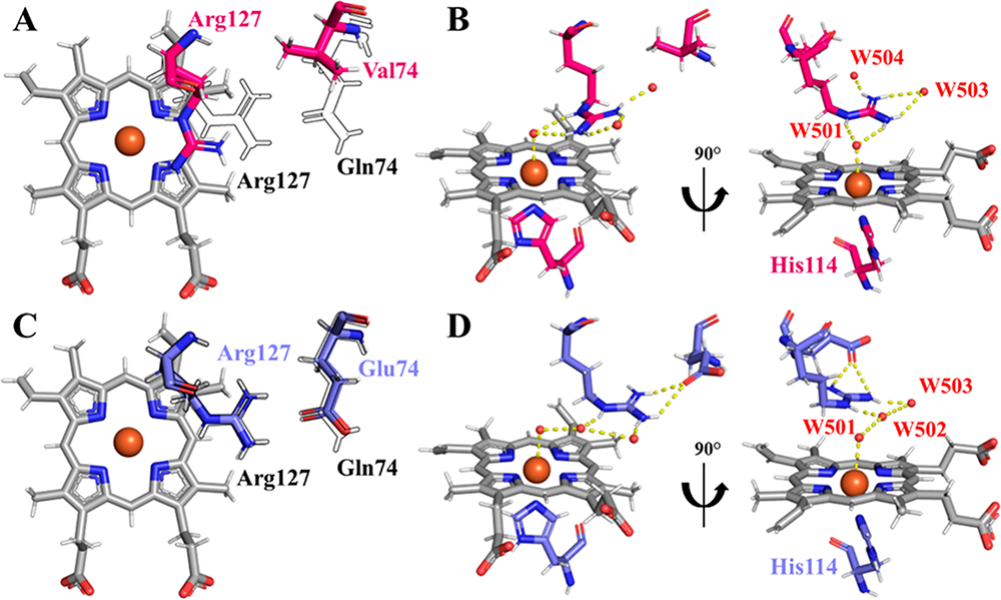
Active site architecture of *C*Cld variants Q74V (pH 8.5) and Q74E (pH 6.5). (A and C) Overlay of the important residues on the distal side (Arg127 and Q/V/E74) of the heme cofactor (gray) in stick representation, with the respective *C*Cld wild-type residues (PDB entry 5MAU) shown as a black outline. (A) Q74V (red) shows a conformational change to the “in” conformation, while Q74E (C, blue) depicts an almost identical conformation compared to the wild-type structure. (B and D) Detailed view of the active site architecture in two different views. Heme iron and waters (W) are shown as orange and red spheres, respectively. Hydrogen bonds are depicted with a yellow dashed line. Highly conserved His114 serves as the fifth heme ligand on the proximal side. This figure was generated using PyMOL (http://www.pymol.org/).

Histidine 114 serves as the fifth heme ligand on the proximal side, with the N_ε_2 atom being 2.15 Å from the heme iron in both structures (Figure 7B,D). On the distal side, a H_2_O molecule (W501) is situated above the heme iron at a distance of 2.6 Å. The conformation of the distal arginine was significantly affected by exchange of Gln74. While in Q74E (pH 6.5) R127 adopts a nearly identical (“out”) conformation as in wild-type *C*Cld^14^ (Figure 7), in Q74V (pH 8.5) it is pointing toward the heme iron (Figure 7). In Q74V (deprotonated), W501 is directly H-bonded to N_ε_ (2.2 Å) and N_η_2 (2.3 Å) of R127 (Figure 7). In Q74E, the distal W501 is hydrogen bonded to a second conserved water molecule (W502, 2.5 Å), which in turn forms an H-bond to the N_ε_ atom (2.0 Å) of R127 in the “out” conformation, which is stabilized by a salt bridge between R127 and E74 (Figure 7). The distances between N_η_1 and N_η_2 of R127 and O_ε_1 of E74 are 2.3 and 2.6 Å, respectively. The second available hydrogen of N_η_2 is further H-bonded to a H_2_O molecule (W503, 2.0 Å), which in addition forms a hydrogen bond to W502. In Q74V, the “in” conformation of R127 leads to altered H-bonds of N_η_1 (2.5 Å) and N_η_2 (2.3 Å) to W503. Furthermore, N_η_1 of R127 is H-bonded to an additional H_2_O molecule (W504), which is not present in wild-type *C*Cld^14^ and Q74E (Figure 7B,D). Interestingly, the architecture of the α-helical loop, which connects the N-terminal and C-terminal ferredoxin-like fold in *C*Cld protomers, is almost identical in the three proteins.

### Kinetics of Chlorite Degradation

Interestingly, the catalytic efficiency (*k*_cat_/*K*_M_) of chlorite degradation of both variants was similar to that of the wild-type protein (Table 4) with a pH optimum around 5. In the pH regime from 5.0 to 9.0, Q74V exhibits the lowest *K*_M_ values, which decrease with an increase in pH (pH 5.0, 47.8 μM; pH 9.0, 19.3 μM). In both wild-type *C*Cld and Q74E, the same pH dependence of *K*_M_ values is observed [wild-type CCld, 127.1 μM (pH 5.0) and 59.2 μM (pH 9.0); Q74E, 327.0 μM (pH 5.0) and 21.0 μM (pH 9.0)]. With all three proteins, the *k*_cat_ values are highest at acidic pH values and decrease with an increase in pH (Table 4). At the pH optimum, the *k*_cat_ values decrease in the following order: Q74E > wild-type *C*Cld > Q74V. It must be mentioned that above 100 μM chlorite (5000-fold excess) irreversible inactivation is observed with all three proteins. At pH >7.0, both Q74V and Q74E are more prone to inactivation compared to wild-type *C*Cld (Figure S7).

**Table 4:** Kinetic Parameters of Wild-Type *C*Cld and Variants Q74V and Q74E at pH 5.0, 7.0, and 9.0^a^

*C*Cld	*K*_M_ (μM)	*k*_cat_ (s^–1^)	*k*_cat_**/***K*_M_ (M^–1^ s^–1^)
pH 5			
wild type	127.1 ± 28.0	475.8 ± 34.7	(3.7 ± 1.2) × 10^6^
Q74V	47.8 ± 4.7	280.2 ± 2.8	(5.9 ± 0.7) × 10^6^
Q74E	327.0 ± 6.6	977.4 ± 34.4	(3.0 ± 0.2) × 10^6^
pH 7			
wild type	43.2 ± 5.2	60.0 ± 0.7	(1.4 ± 0.2) × 10^6^
Q74V	18.8 ± 3.5	50.8 ± 0.4	(2.7 ± 0.5) × 10^6^
Q74E	53.8 ± 13.1	37.2 ± 1.1	(6.9 ± 2.0) × 10^5^
pH 9			
wild type	59.2 ± 20.1	33.4 ± 1.5	(5.6 ± 2.5) × 10^5^
Q74V	19.3 ± 6.0	43.0 ± 0.7	(2.2 ± 0.8) × 10^6^
Q74E	21.0 ± 11.2	18.91 ± 0.5	(0.9 ± 0.7) × 10^6^

a Chlorite degradation was monitored polarographically.

## Discussion

Functional Clds possess a conserved distal arginine in an otherwise hydrophobic active site pocket, which in all crystal structures of clade 2 Clds published thus far (including the model enzyme *C*Cld) is in the “out” conformation and forms a hydrogen bond to Q74. Glutamine 74 is located on the α-helical loop that connects the N- and C-terminal domains of a protomer.^11,14^ In clade 1 Clds, a hydrogen-bonding partner is missing due to different conformations of this loop region (Figure 2).^7−9^*C*Cld variant Q74V was designed to mimic this setting in clade 1 Clds, whereas the design of *C*Cld variant Q74E aimed to study the impact of arresting R127 in a salt bridge with E74.

In the crystal structure of Q74V determined at pH 8.5, R127 adopts the “in” conformation and directly interacts with the deprotonated distal W501 (Figure 7), which is different to the orientation of R127 in X-ray and neutron structures of wild-type *C*Cld determined at pH 6.5, 8.5, and pH 9.0 as well as those of complex structures with fluoride and thiocyanate.^14^ In all of these wild-type *C*Cld data sets,^14^ the R127 side chain is positioned away from the heme moiety. This “out” conformation is stabilized by hydrogen bonding between N_η_1 of R127 and O_ε_1 of Q74.^14^ Because the p*K*_a_ for the alkaline transition in Q74V has been determined to be 7.41 compared to 8.11 for wild-type *C*Cld (Figure 3), it is reasonable to assume that its (more flexible) guanidinium group promoted the deprotonation of W501. Both the decrease in the p*K*_a_ of the alkaline transition and the X-ray structure suggest a higher flexibility in solution of R127 in Q74V compared to the wild-type protein. The opposite trend is visible for variant Q74E. At pH 6.5, its active site architecture strongly resembles that of wild-type *C*Cld with the exception that R127 is arrested in a salt bridge with E74 (Figure 7). As a consequence, its guanidinium group is not able to stabilize the conjugated base of W501 and the p*K*_a_ of the alkaline transition is significantly increased to a p*K*_a_ of 9.33 (Figure 3). These findings clearly demonstrate experimentally that (i) the distal arginine in wild-type Clds is flexible, (ii) its dynamics is influenced by its remote H-bonding partner, and (iii) in Q74V (mirroring clade 1 enzymes) this flexibility is more pronounced than in wild-type *C*Cld.

Most interestingly, these differences in the conformational dynamics of R127 had a strong impact on the thermal stability of the heme cavity. In principle, the thermal stability of pentameric clade 1 Clds (i.e., *Nd*Cld) is significantly higher than that of dimeric clade 2 Clds like *C*Cld. Thermal unfolding of clade 1 enzymes follows a simple two-state transition, suggesting a cooperative process, in which separation of subunits, monomer melting, and release of the heme cofactor occur simultaneously.^41^ By contrast, dimeric Clds, including *C*Cld, follow a non-two-state unfolding pathway with two consecutive unfolding events. The differences in N-terminal domain architecture and protomer interactions^6−8,11,14^ have been proposed to be responsible for these observations.^38^ As typical clade 2 Clds, wild-type *C*Cld and variants Q74V and Q74E show two endotherms in DSC with the second unfolding event (i.e., melting of the overall secondary structures) occurring at similar *T*_m_2 values (∼58 °C) in the three proteins. By contrast, the first unfolding event strongly depends on the H-bonding network and conformational dynamics of R127. At pH 5.0, the *T*_m_1 values increase in the following order: Q74V (38.3 °C) < wild-type *C*Cld (45.8 °C) < Q74E (50.9 °C). As suggested by thermal ECD data in the visible region, at these temperatures the active site unfolds and heme is released (Table S4). In the case of Q74E, the salt bridge of R127 with E74, which is located at the α-helical loop (Figures 2 and 7), stabilizes the heme cavity by increasing the conformational stability of the α-helical loop (Figure 2). Disruption of the salt bridge at 50.9 °C (pH 5.0) seems to initiate unfolding of the loop and opening of the heme cavity in Q74E.

At acidic and neutral pH, the EPR spectra of the three proteins do not show significant differences (Figure 4). At pH 7.0, the high-spin ferric heme signal of Q74V still accounts for 97% of the spectrum, whereas the spectrum of Q74E already shows some minor low-spin signals (Figure S4). This may seem partially inconsistent with the p*K*_a_ values obtained from UV–vis spectroscopy (Figure 3 and Figure S2); however, in EPR, the effects of freezing and formation of artifacts at cryogenic temperatures have to be taken into account.^42,43^ In any case, the spectroscopic findings clearly demonstrate that at the pH optimum of chlorite degradation activity (i.e., pH 5) (i) the heme coordination in the three proteins is similar, i.e., the geometry of the paramagnetic center is not substantially perturbed, and consequently (ii) the impact of the conformational dynamics of R127 is small.

In the alkaline pH regime, the geometries of the heme pockets of wild-type *C*Cld and the variants Q74V and Q74E change. This includes changes in the rhombicity of the EPR signals of the high-spin components and formation of new low-spin species whose EPR features are consistent with the ones of hydroxo-ligated forms described for other chlorite dismutases.^12,38−40^ The attribution of these signals to OH^–^ adducts is additionally supported by resonance Raman spectroscopy studies, which highlighted the presence of a Fe–OH bond in the alkaline forms of several chlorite dismutases from both clade I and clade II.^14,17,18,44^ In wild-type *C*Cld (Figure 4A), the high-spin components with an axial **g**^eff^ tensor, which dominate the spectrum at acidic and neutral pH (HS1 and HS2), progressively convert to a single and well-resolved rhombic species (HS3) at pH 10.0 (Figure 4A). This reflects a different symmetry and altered geometry of the active site pocket most probably by repositioning of R127 and its H-bonding network. This is further corroborated by the analysis of the low-spin species identified at pH 10.0 in both *C*Cld and *Nd*Cld (Tables 1 and 2, Table S2, and Figures S3 and S5). LS1-alkaline (*g*_*x*_ = 1.87, *g*_*y*_ = 2.20, *g*_*z*_ = 2.53) and LS2-alkaline (*g*_*x*_ = 1.83, *g*_*y*_ = 2.19, *g*_*z*_ = 2.63) are the principal contributions in the high-pH spectrum of wild-type *C*Cld, while a third species (LS3-alkaline: *g*_*x*_ = 1.80, *g*_*y*_ = 2.27, *g*_*z*_ = 2.75) contributes to the spectrum to a lesser extent. These components have *g* values similar to those of OH^–^ adducts of other heme proteins, such as cytochrome *c* peroxidase,^45^ human hemoglobin,^46^ horse heart myoglobin,^46^ and *Caenorhabditis elegans* globin-33.^47^ Nicoletti et al. showed that at high pH hemoglobin from *Thermobifida fusca* forms two hydroxo complexes with different sets of *g* values,^48^ which they explained with the OH^–^ group being hydrogen-bonded to different extents to nearby residues. In the case of hemoglobin, the component with broader features was assigned to a strong His/OH^–^ coordination. The differences in the H-bonding network most probably result in a different electron density distribution between the axial ligand(s) and the porphyrin, which primarily determines the magnitude of the tetragonal parameter according to crystal field theory.^49,50^ Indeed, as nicely exemplified by Svistunenko et al.,^46^ the more unpaired electron density is on the heme iron, the more anisotropic is the **g** tensor of the low-spin signal. Therefore, it is possible that the LS contributions in the spectrum of wild-type *C*Cld derive from the dynamics of R127, which allows the hydroxide group to attend different H-bonding networks.

In contrast to wild-type *C*Cld, the EPR spectrum at pH 10 in both Q74E and Q74V is dominated by an LS2-alkaline contribution with larger *g* anisotropy than the LS1-alkaline component, which suggests a higher spin density on the heme (Figure S5). This points to a different H-bonding dynamics of the hydroxide group due to the altered flexibility of R127. Interestingly, the main hydroxide adduct in *Nd*Cld is characterized by *g* values similar to those of LS2-alkaline (Figure S3), suggesting that the heme pocket of the pentameric Cld is very similar to that of *C*Cld Q74V, which was designed as a model for clade 1 Clds.

The EPR spectra of both *Nd*Cld and *C*Cld show features of low-spin states even at pH ≤7; however, their interpretation is not trivial. In the case of *Nd*Cld, two broad low-spin contributions (Table S3, LS1′ and LS2′) have *g* values consistent with other low-spin species previously observed in several EPR studies of chlorite dismutases at neutral pH. While in certain cases their origin could not be determined,^12^ in the work of De Schutter et al.^38^ it was demonstrated that some of these species may represent “ready” states still accessible to the binding of external molecules. Moreover, in a comparative study of native and recombinant chlorite dismutase from *Ideonella dechloratans*, it was shown that a low-spin signal consistent with a bis-histidine coordination was present in only the recombinant form.^51^ In another work, the occurrence of the typical features of an imidazole adduct was explained as artifacts from the protein purification procedure.^6^ In the case of *Nd*Cld, no imidazole was used during purification; therefore, this explanation can be ruled out. Furthermore, the predominance of the LS1′ signal in *Nd*Cld samples prepared at different pH values and in different buffers makes it unlikely to be due to the binding of an external molecule present in the medium and suggests a strong coordination from a ligand that cannot be easily displaced. For these reasons, the nature of the LS species remains elusive. In the case of *C*Cld, the *g* values of LS1 (*g*_*x*_ = 1.66, *g*_*y*_ = 2.28, *g*_*z*_ = 2.81) found at neutral and acidic pH are unique and (to the best of our knowledge) have never been reported before in Clds. This species is characterized by features that are consistent with neither imidazole–heme complexes^6,12,52^ nor the OH^–^ adducts formed at high pH, as shown above. However, these values present some similarities with the hydroxide complexes found in other heme protein samples at non-alkaline pH.^43,46^

Both UV–vis and EPR data have demonstrated that modulation of the H-bonding network of R127 in wild-type *C*Cld and the two variants do not substantially perturb the spin state of the heme iron in the ferric resting state in solution in the acidic and neutral pH region. This fits with observation that at a pH optimum (i.e., pH 5.0) the catalytic efficiency of wild-type *C*Cld and of the two variants is in the range of 3.0 × 10^6^ to 5.9 × 10^6^ M^–1^ s^–1^, very similar to those of previously studied chlorite dismutases from clade 2 and most of clade 1 Clds (Table 4).^8−14^ Because even arresting R127 in a salt bridge (Figure 7) had almost no effect on the catalytic efficiency, this suggests that the impact of the conformational dynamics of R127 on catalysis is relatively small.

At the pH optimum and neutral pH, the *K*_M_ values decrease in the following order: Q74E > wild-type *C*Cld > Q74V. This mirrors the hierarchy of the thermal stability of the heme cavity. By contrast, the *k*_cat_ values follow the opposite order at pH 5.0. On the basis of the findings presented here and recently published data on both clade 1 and clade 2 Clds, we propose the following functional role(s) of the only charged amino acid in the distal cavity of *C*Cld.

The reaction cycle of dimeric *C*Cld is initiated by binding of the anionic substrate (p*K*_a_ = 1.72) to the ferric heme center. At first sight, at the pH optimum in *C*Cld the flexibility of the arginine seems to facilitate this reaction to some extent, because the *K*_M_ of Q74E was significantly higher compared to that of wild-type *C*Cld. The differences in *K*_M_ values are less pronounced at higher pH values. In pentameric *Nd*Cld, it has been demonstrated that exchange of R173 with either alanine or glutamine had an only weak effect on the respective *K*_M_ values, suggesting that its role in chlorite binding is negligible.^9^ This was further supported by MD data, which showed that R173 does not play a role in shuttling the anion into the active site. It rather followed the charged molecules and changes its orientation (“in” vs “out” accordingly), suggesting that its role in the initial reaction step is chlorite recognition only.^53^ By contrast, in clade 1 Cld from *Dechloromonas aromatica* exchange of R183 with alanine increased the *K*_M_ from 0.2 to 14.6 mM.^17^

Differences between clade 1 and clade 2 Clds may be related to the differences between the α-helical loop that connects the N- and C-terminal domains of Cld protomers. Due to its location at the entrance to the heme cavity, it governs the accessibility to the heme cavity. In *C*Cld variant Q74V and clade 1 *C*Clds, the interaction between the distal arginine and the loop is small, whereas in wild-type *C*Cld, a hydrogen bond between R127 and Q74 is formed. By contrast, Q74E increases the rigidity of the loop (as reflected by the DSC data) and thus decreases its dynamics, which seems to be important for chlorite accession and binding. Principally, chlorite binding should occur spontaneously and independent of arginine as demonstrated for *Nd*Cld. This study also rules out that the alkaline transition is responsible for the pH dependence of chlorite degradation activity. Despite the fact that the p*K*_a_ of the alkaline transition is significantly different in the three proteins due to differences in the flexibility of R127, the pH dependence of the catalytic efficiency was similar in wild-type *C*Cld and the two variants.

Elucidation of HS and LS complexes of *C*Cld studied at pH 6.5 demonstrated that addition of a ligand, irrespective of its nature, eliminates W501 but not W502 from the heme cavity and keeps R127 in the “out” conformation.^14^ This also applies for the LS ligand isothiocyanate, which may act as a model for chlorite.^14^ The crystal structure of the *C*Cld–isothiocyanate complex suggests that the substrate is hydrogen bonded to W502 and R127 in the “out” conformation. Immediately after this binding event, chlorite is converted to chloride and dioxygen. It has been demonstrated with *Nd*Cld that in this reaction the distal R173 is important but not fully essential. Exchange of R173 by either alanine or glutamine led to significant decreases in the respective turnover numbers, i.e., ∼6.5% (R173A) and ∼5.4% (R173Q).^9^ In addition to the decreased *k*_cat_ values, the *Nd*Cld R173 variants were also more prone to inactivation than the wild-type enzyme by oxidative modification of the heme cofactor and the protein.^9,16^ In any case, the impact of the conformational dynamics of R127 in *C*Cld on the cleavage reaction seems to be small because at the pH optimum Q74E exhibits the highest turnover number. Apparently, R127 in the arrested “out” conformation is still able to support the homolytic or heterolytic cleavage of chlorite.^1,2,6−9,12,14^

Independently of the mechanism of chlorite cleavage, the resulting transient intermediates (either Compound I and hypochlorite or Compound II and chlorine monoxide) must be kept in the reaction sphere for the following rebound step. It has been demonstrated that the bond cleavage in chlorite occurs very fast and independent of pH.^14^ Thus, it was hypothesized that the following rebound step is responsible for the observed pH dependence.^14^ In this step, the guanidinium group seems to play an important role by keeping the reaction partners in place. Computational studies suggest that in the rebound step the transient intermediates (either hypochlorite or chlorine monoxide) must rotate. This rotation requires rearrangements of H-bonds with the arginine to support O–O bond formation. Finally, the H-bond between the arginine and the two oxygen atoms needs to rearrange again to the terminal chlorine atom.^54^ The data presented here clearly suggest that R127, arrested in a salt bridge with E74 and thus in the “out” conformation, can still participate in these proposed reaction steps.

In addition to the decrease in the rate of chlorite degradation, Clds become inactivated at alkaline pH and this inactivation reaction is promoted in the absence of the arginine. This is also the case for *C*Cld.^13^ It was proposed that at alkaline pH values, where O–O bond formation apparently becomes inefficient, intermediates (hypochlorite or chlorine monoxide) escape from the reaction sphere, which oxidatively modify both the heme cofactor and the protein. Increasing the level of irreversible inhibition with an increase in pH is also seen in this study. Here the role of the dynamics of R127 remains elusive because upon exchange of glutamine with either valine or glutamate the extent of inactivation was slightly higher compared to that of the wild-type protein (Figure S7).

In summary, we have demonstrated that the conformational dynamics of the catalytic arginine can be modulated by its remote H-bonding network. This is underlined by the presented high-resolution crystal structures and the impact of the flexibility of R127 on the alkaline transition. Formation of the LS hydroxide complex is shown by UV–vis and EPR spectroscopies, and the calculated p*K*_a_ values strongly depended on the flexibility of R127. By contrast, the effect on the catalytic efficiency in the pH regime between 5.0 and 9.0 was small, suggesting that R127 in the “out” conformation can support chlorite cleavage and keep the transient intermediates in place. Most interestingly, the strongest impact of the exchange R127 was on the thermal stability and the *K*_M_ values at the pH optimum. This seems to be related to the fact that R127 is H-bonded to the Cld-typical (flexible) α-helical loop in *C*Cld that might act as a gatekeeper for the active site. Arresting this loop in the salt bridge (Q74E) significantly increases the thermal stability of the heme cavity but at the same time decreases the loop dynamics that is necessary for chlorite to enter the active site and bind to the heme iron.

## Supporting Information Available

Multiple-sequence alignments of clade 1 and 2 Clds (Table S1), detailed information about primers (Table S2), EPR simulation parameters (Table S3), and EPR spectra of *Nd*Cld, wild-type *C*Cld, and variants Q74V and Q74E at various pH values (Figures S3–S5, respectively), data about thermal stabilities at various pH values (Table S4), electron density maps of Q74V and Q74E (Figure S6), and kinetic parameters (Figure S7) (PDF)

## Accession Codes

Cld from *Cyanothece* sp. PCC7425 (*C*Cld), UniProt entry B8HNS6, PDB entry 5MAU; Cld from *Candidatus* “*Nitrospira defluvii*” (*Nd*Cld), UniProt entry B3U4H7, PDB entry 3NN1.

## Abbreviations

Cld: chlorite dismutase
*C*Cld: chlorite dismutase from *Cyanothece* sp. PCC7425
CW: continuous wave
*D*: tetragonal
DSC: differential circular calorimetry
*E*: rhombic
ECD: electronic circular dichroism
EPR: electron paramagnetic resonance
HS: high-spin
LS: low-spin
*Nd*Cld: chlorite dismutase from *Candidatus* “*Nitrospira defluvii*”
PDB: Protein Data Bank
ZFS: zero-field splitting.
