# Supplementary material for: Arresting the Catalytic Arginine in Chlorite Dismutases: Impact on Heme Coordination, Thermal Stability, and Catalysis
Source: Biochemistry. 2021 Feb 15;60(8):621–34. doi: 10.1021/acs.biochem.0c00910 (PMC7931450; doi:10.1021/acs.biochem.0c00910)
Supplement: Supplementary file 1 — bi0c00910_si_001.pdf [file bi0c00910_si_001.pdf]

ASSOCIATED CONTENT

Arresting the catalytic arginine in chlorite dismutases – Impact on heme coordination, thermal stability and catalysis

Daniel Schmidt[1], Ilenia Serra[2**], Georg Mlynek[3], Vera Pfanzagl[1], Stefan Hofbauer[1], Paul G. Furtmüller[1], Kristina Djinović-Carugo[3,4], Sabine Van Doorslaer[2*], Christian Obinger[1*]**

[1]Department of Chemistry, Institute of Biochemistry, University of Natural Resources and Life Sciences, Vienna, Muthgasse 18, A-1190 Vienna, Austria

[2]BIMEF Laboratory, Department of Chemistry, University of Antwerp, Belgium.

[3]Department of Structural and Computational Biology, Max Perutz Labs, University of Vienna, A-1030 Vienna, Austria

[4]Department of Biochemistry, Faculty of Chemistry and Chemical Technology, University of Ljubljana, Večna pot 5, SI-1000 Ljubljana, Slovenia

** Both authors contributed equally to this manuscript

*Corresponding authors:

Christian Obinger: Phone: +43-47654-77273, Fax: +43-1-47654-77250. E-mail: christian.obinger@boku.ac.at

Sabine Van Doorslaer: Phone: +32-32652461, E-mail: sabine.vandoorslaer@uantwerpen.be

Table S 1. Multiple sequence alignments of Clade 1 & 2 Clds. Note that in all clade 2 Clds R127 is H-bonded to Q74 located on a flexible α-helical loop (Figure 2), which connects the N-terminal and the C-terminal ferredoxin-like domains. By contrast, in clade 1 Clds the corresponding residue (Q123 in *Nd*Cld, but S or T in other clade 1 Clds) turns away from R173 due to a different conformation of this loop (Figure 2). As a consequence, a valine comes close to R173 in *Nd*Cld.

Clade 1	118	119	120	121	122	123	124	125	126	127	128	155	156	157	158	159	160	161	162	163	164	165	166	167	168	169	170	171	172	173	174	175	176	177	178
Nitrospira defluvii	M	K	T	E	L	Q	V	N	G	E	S	A	L	M	Q	E	H	T	Q	A	A	L	P	Y	L	K	T	V	K	R	K	L	Y	H	S
Magnetospirillum magnetotacticum	L	N	S	G	L	S	S	A	S	Y	T	K	E	M	E	V	H	T	Q	P	T	L	Q	Y	L	V	N	V	K	R	K	L	Y	H	S
Ideonella dechloratans	L	N	K	G	L	S	G	A	T	Y	A	K	E	M	E	T	H	T	L	P	T	L	P	F	L	V	N	V	K	R	K	L	Y	H	S
Dechloromonas aromatica	L	N	A	G	L	S	S	A	T	Y	S	K	E	M	E	V	H	T	T	P	T	L	A	Y	L	V	N	V	K	R	K	L	Y	H	S
Azospira oryzae	L	N	A	G	L	S	S	A	T	Y	S	K	E	M	E	V	H	T	T	P	T	L	A	Y	L	V	N	V	K	R	K	L	Y	H	S
Dechlorosoma suillum	L	N	A	G	L	S	S	A	T	Y	S	K	E	M	E	V	H	T	T	P	T	L	A	Y	L	V	N	V	K	R	K	L	Y	H	S
Dechloromonas hortensis	L	N	A	G	L	S	S	A	T	Y	S	K	E	M	E	V	H	T	T	P	T	L	A	Y	L	V	N	V	K	R	K	L	Y	H	S
Dechloromonas agitata	L	N	A	G	L	T	G	A	T	Y	R	K	E	M	E	T	H	T	L	P	T	L	A	N	L	V	N	V	K	R	K	L	Y	H	S

Clade 2	69	70	71	72	73	74	75	76	77	78	79	109	110	111	112	113	114	115	116	117	118	119	120	121	122	123	124	125	126	127	128	129	130	131	132
Cyanothece sp. PCC 7425	A	L	Q	A	V	Q	P	M	L	N	R	E	R	E	S	H	H	T	A	V	G	L	E	Y	L	P	G	V	A	R	R	L	L	H	C
Nitrobacter winogradskyi	A	L	T	S	V	Q	A	G	L	G	R	E	D	K	S	H	H	I	A	A	S	L	K	Y	L	P	A	I	A	R	Q	L	Y	H	C
Bradyrhizobium japonicum	Q	L	T	A	V	Q	A	G	L	G	R	E	D	K	S	H	H	I	A	S	S	L	R	F	L	P	A	I	A	R	Q	L	Y	H	S
Klebsiella pneumoniae	R	L	V	A	K	Q	P	S	L	G	R	E	E	Q	S	R	H	I	H	I	G	L	Q	Y	L	P	A	V	A	R	R	L	H	H	C
Escherichia coli	R	L	V	A	K	Q	P	S	L	G	R	E	E	Q	S	R	H	I	H	I	G	L	Q	Y	L	P	A	V	A	R	R	L	H	H	C
Pseudomonas aeruginosa	R	L	V	A	K	Q	P	S	L	G	R	E	E	Q	S	R	H	I	H	I	G	L	Q	Y	L	P	A	V	A	R	R	L	H	H	C
Acinetobacter baumannii	E	I	V	A	K	Q	Q	G	L	A	R	E	-	K	S	K	H	I	Q	I	G	L	N	Y	L	P	A	V	A	R	K	L	H	H	C

Table S2. Primers used for cloning and site directed mutagenesis

Name	Primer
**Exchange of affinity tag**	
pET52b_Strep2HIS_fwd	5’-ACCATCATCATCATCACGGACTTGAAGTCCTCTTTCAGGGACCC-3’
pET52b_Strep2HIS_rev	5’-TCCGTGATGATGATGATGGTGGCTTGCCATGGTATATCTCCT-3’
**Site directed mutagenesis**	
Q74V_fwd	5’-TCTGCAGGCTGTGGTGCCGATGCTGAACC-3’
Q74V_rev	5’-GGTTCAGCATCGGCACCACAGCCTGCAGA-3’
Q74E_fwd	5’-GCTCTGCAGGCTGTGGAACCGATGCTGAACCGC-3’
Q74E_rev	5’-GCGGTTCAGCATCGGTTCCACAGCCTGCAGAGC-3’

Table S3. EPR simulation parameters of wild-type *Nd*Cld at different pH values (errors: *g* values ±0.01, *E/D* ratio ±0.001, contribution ±1%)

	Species	g_x^{eff}	g_y^{eff}	g_z^{eff}	g_x	g_y	g_z	***E/D***	**%**
pH 5.5	HS1’	5.87	5.87	1.98	1.96	1.96	1.98	< 0.001	< 1
	HS2’	6.17	5.52	1.99	1.95	1.95	2.00	0.014	21
	HS3’	6.47	5.28	1.98	1.96	1.96	2.00	0.026	2
	LS1’				1.24[a]	2.11	3.15	-	71
	LS2’				1.43[a]	2.25	2.98	-	5
pH 7	HS1’	5.89	5.89	1.98	1.97	1.97	1.98	0.002	1
	HS2’	6.17	5.52	1.99	1.95	1.95	2.00	0.014	20
	HS3’	6.47	5.29	1.99	1.96	1.96	2.00	0.025	2
	LS1’				1.24[a]	2.11	3.16	-	71
	LS2’				1.36[a]	2.26	3.01	-	6
pH 10	HS4’	6.18	5.75	2.00	1.99	1.99	2.00	0.009	7
	HS5’	6.35	5.54	1.99	1.98	1.98	2.00	0.017	2
	LS1’				1.27[a]	2.10	3.16	-	59
	LS2’				1.22[a]	2.28	3.05	-	6
	LS1’-alkaline				1.87	2.28	2.55	-	1
	LS2’-alkaline				1.82	2.19	2.65	-	21
	LS3’-alkaline				1.80	2.28	2.60	-	4

[a] *values were calculated assuming the sum of the squares of the g principal values is ~ 16*

Table S4. Thermal stability of wild-type (wt) *C*Cld and the variants Q74V and Q74E followed by differential scanning calorimetry (DSC) and electronic circular dichroism (ECD) in the far-UV and visible region at pH 5, 7 and 9, respectively.

pH 5				
**Temp [°C]**	**Method**	***C*Cld Q74V**	***C*Cld wt**	***C*Cld Q74E**
T_m1	DSC	38.3 ± 0.1	46.2 ± 0.4	50.3 ± 0.6
	ECD (visible)	40.1 ± 0.2	55.5 ± 0.3	57.0 ± 1.0
T_m2	DSC	57.7 ± 0.8	58.2 ± 0.3	59.1 ± 0.6
	ECD (far UV)	56.9 ± 0.2	57.3 ± 0.1	57.6 ± 0.1
**pH 7**				
**Temp [°C]**	**Method**	***C*Cld Q74V**	***C*Cld wt**	***C*Cld Q74E**
T_m1	DSC	44.8 ± 0.3	51.0 ± 0.5	54.7 ± 0.4
	ECD (visible)	45.4 ± 0.4	48.7 ± 0.2	52.5 ± 0.2
T_m2	DSC	62.8 ± 0.5	63.2 ± 0.4	64.4 ± 0.1
	ECD (far UV)	63.0 ± 0.2	63.2 ± 0.2	64.0 ± 0.3
**pH 9**				
**Temp [°C]**	**Method**	***C*Cld Q74V**	***C*Cld wt**	***C*Cld Q74E**
T_m1	DSC	39.8 ± 0.1	45.1 ± 0.1	45.2 ± 0.3
	ECD (visible)	40.1 ± 0.1	44.5 ± 0.1	44.4 ± 0.2
T_m2	DSC	60.9 ± 0.3	61.0 ± 0.3	61.8 ± 0.4
	ECD (far UV)	57.2 ± 0.2	57.2 ± 0.3	58.0 ± 0.3

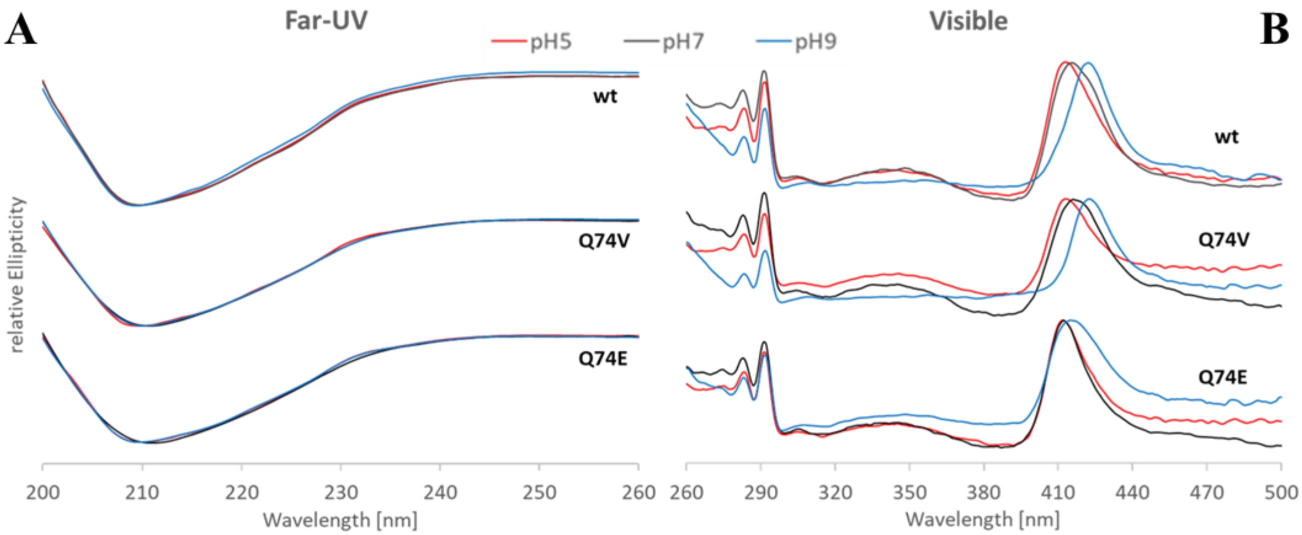

Figure S1. pH-dependent electronic circular dichroism spectra of *C*Cld variants. Overlay of different 10 µM wild-type CCld and the variants Q74V and Q74E spectra in the far UV (A) and visible (B) region. Spectra were obtained at pH 5.0 (red; 5 mM citrate-phosphate buffer), pH 7.0 (black; 5 mM phosphate buffer) and pH 9.0 (blue; 5 mM borate-phosphate buffer).

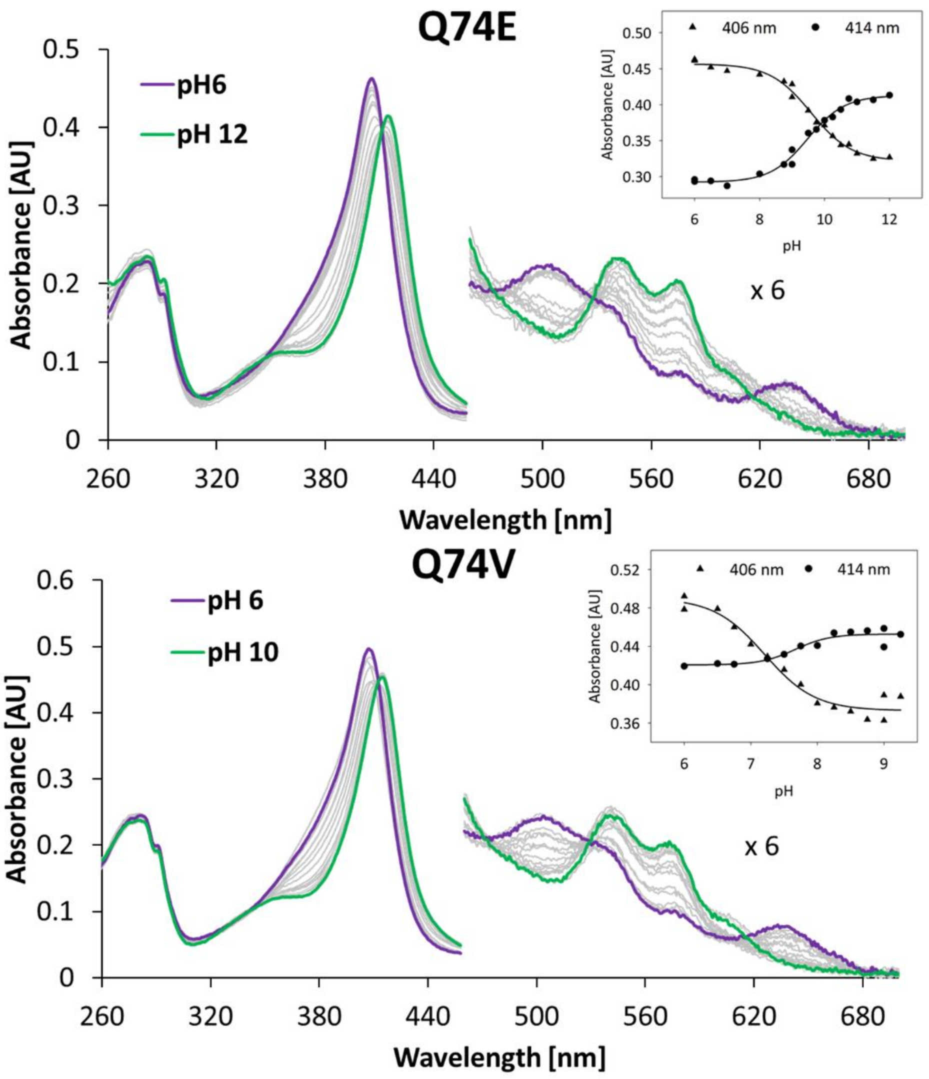

Figure S2. pH dependent UV-vis spectral changes of *C*Cld variants Q74V and Q74E. UV-vis spectra of *C*Cld variants Q74E and Q74V, in the pH range of pH 6.0 - 12.0 and pH 6.0 – 10.0, respectively. Enzyme concentration: 5 µM. For better visualization of the CT and Q bands, the spectra in the wavelength range between 460 nm and 700 nm are magnified 6-fold. Insets show increase and decrease with changing pH at 406 nm and 414 nm, respectively. Furthermore, the sigmoidal fit is shown as black line.

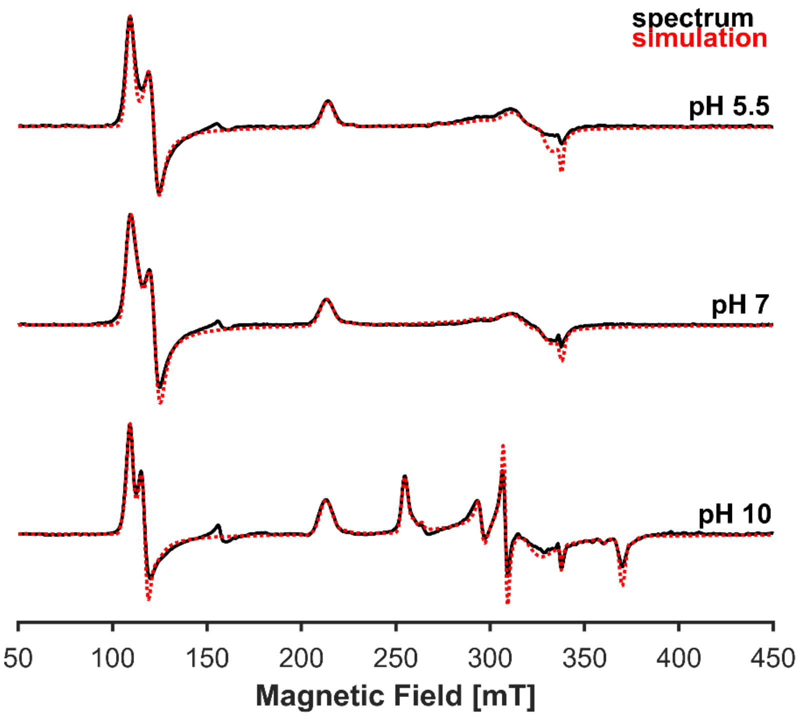

Figure S3. EPR spectra of wild-type *Nd*Cld at acidic (top), neutral (middle) and alkaline pH (bottom). Samples were prepared in 50 mM MES buffer, pH 5.5, 50 mM HEPES buffer, pH 7, and 50 mM borate buffer pH 10; a microwave power of 5 mW was applied to record the spectra. Solid black: experimental spectrum, dashed red: simulation.

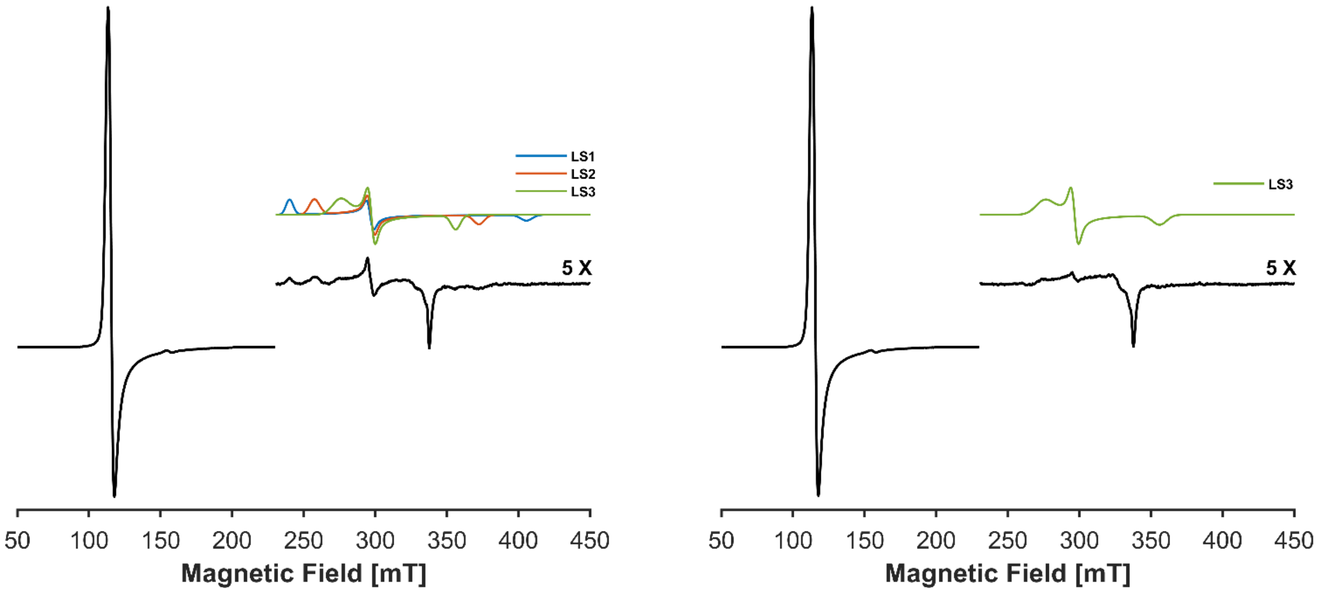

Figure S4. **CW X-band EPR spectra of Q74E (left) and Q74V (right) variants of *C*Cld, at neutral pH.** Samples were prepared in 50 mM phosphate buffer, pH 7.0; a microwave power of 2 mW was applied to record the spectra. For clarity, insets of the high-field region are 5x-magnified and single low-spin species obtained from simulations are shown according to color legend.

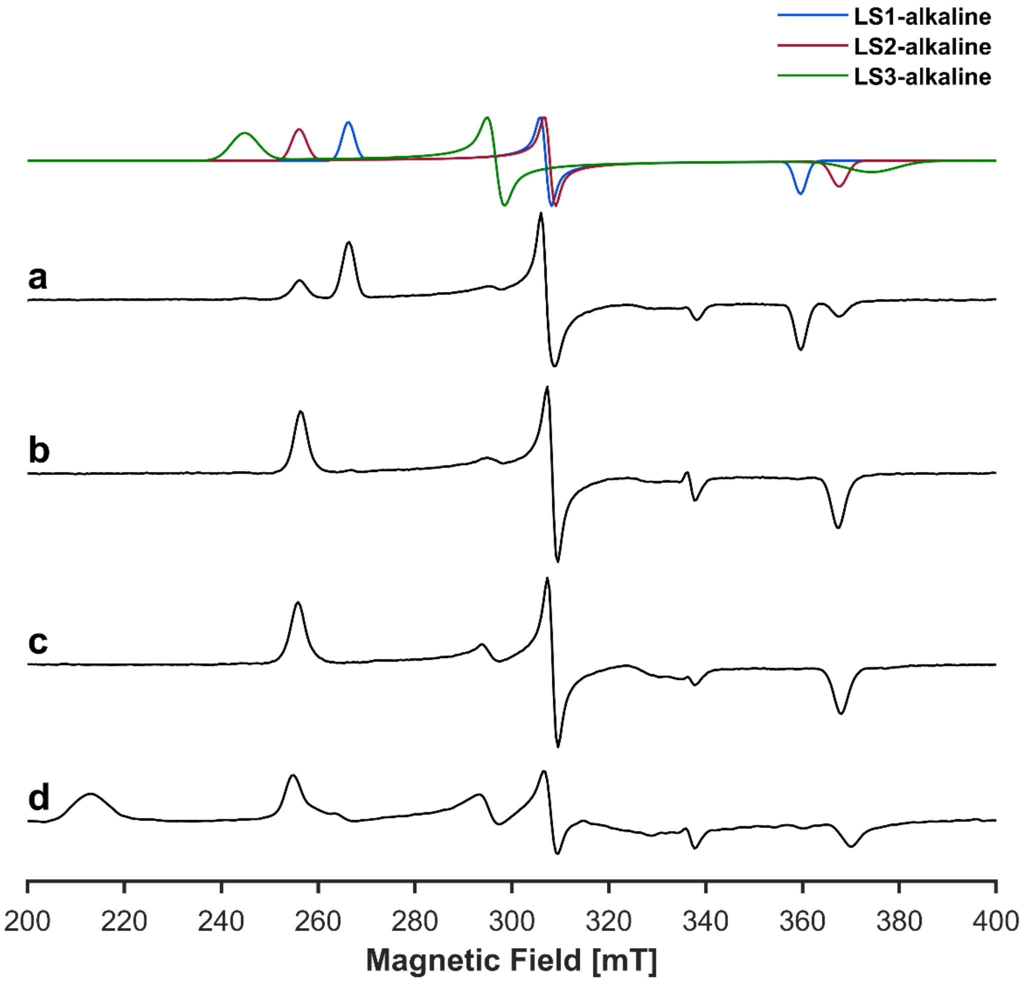

Figure S5. **EPR spectra (low-spin region only) of a) wild-type *C*Cld, b) Q74E, c) Q74V and d) *Nd*Cld in 50 mM borate buffer, pH 10**. Solid black: experimental spectrum; single species obtained from simulations of wild-type *C*Cld are shown according to color legend. The broad feature at ~ 210 mT in the spectrum of *Nd*Cld (trace **d**) arises from the contribution of the low-spin species already observed at neutral pH (LS1' and LS2').

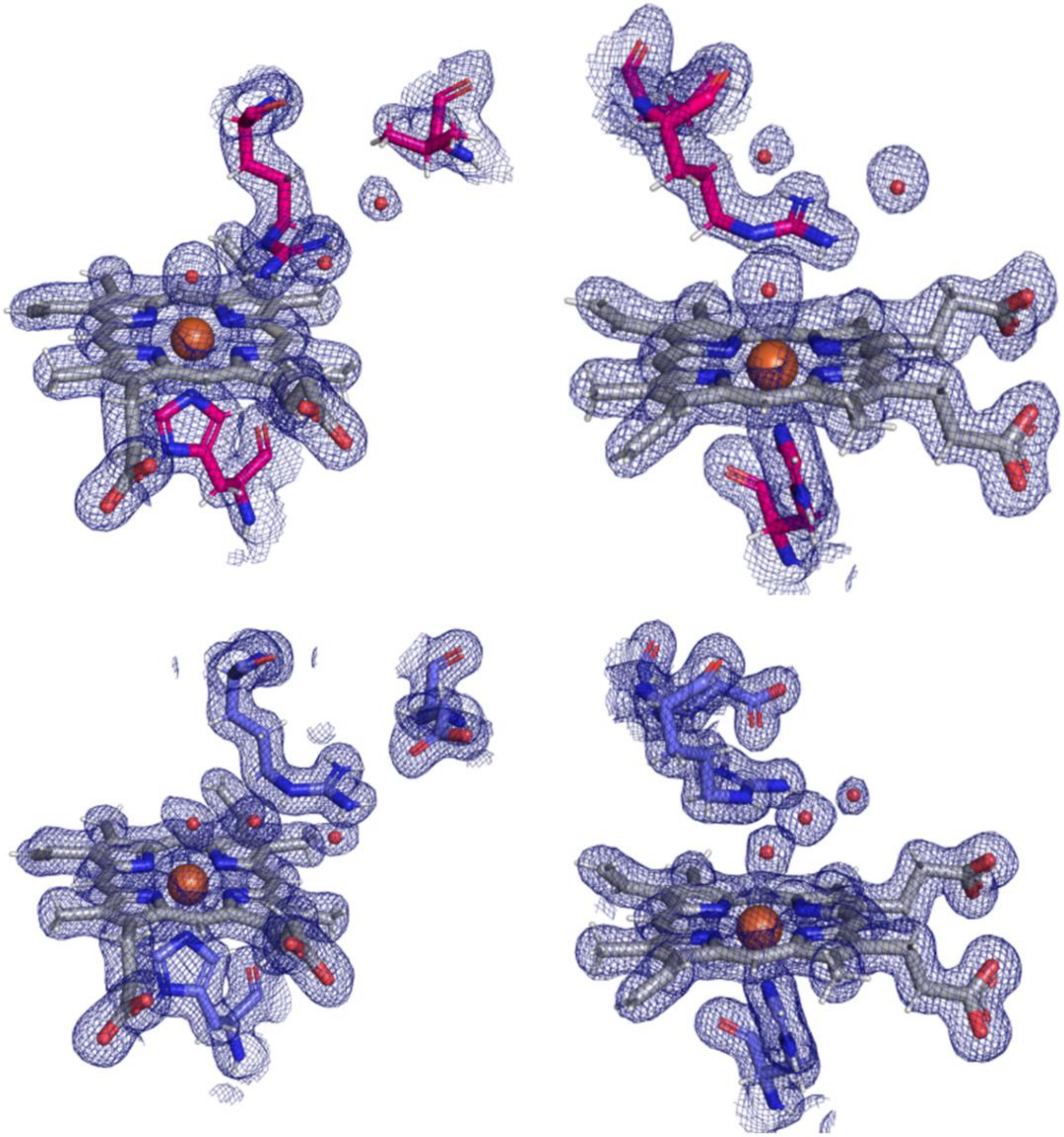

Figure S6. Active site architecture of *C*Cld variants Q74V (red) and Q74E (blue) with $2F_o$-F_c electron density maps contoured at σ = 1.5. Important residues of the active site of *C*Cld variants and the heme *b* cofactor (grey) are shown in stick representation. The heme iron and relevant water molecules are depicted as orange and red spheres, respectively. $2F_o$-F_c electron density maps (blue meshes) were contoured at σ = 1.5 using PyMOL (http://www.pymol.org/).

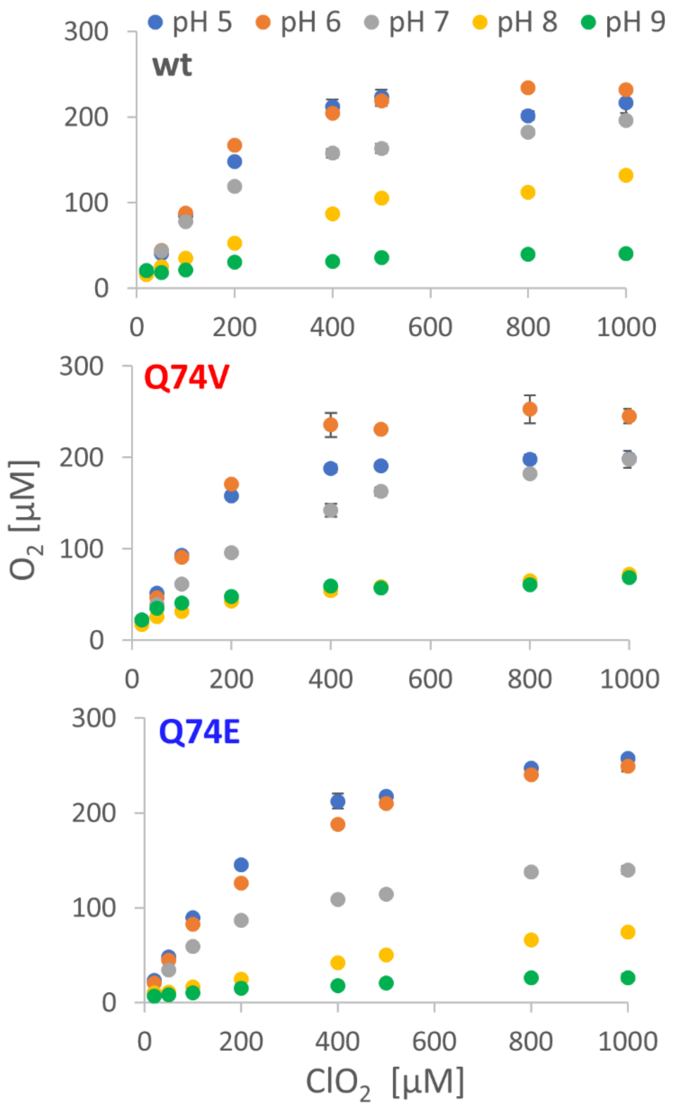

Figure S7. Total produced O_2 using chlorite concentrations ranging from 20-1000 µM chlorite. Enzyme concentration: 20 nM. The measurements were performed at different pH values (pH 5.0-6.0: 50 mM citrate phosphate buffer; pH 7.0-9.0: 50 mM phosphate buffer).
